# Expanded Diversity and Phylogeny of *mer* Genes Broadens Mercury Resistance Paradigms and Reveals an Origin for MerA Among Thermophilic Archaea

**DOI:** 10.3389/fmicb.2021.682605

**Published:** 2021-06-23

**Authors:** Christos A. Christakis, Tamar Barkay, Eric S. Boyd

**Affiliations:** ^1^Department of Microbiology and Immunology, Montana State University, Bozeman, MT, United States; ^2^Department of Biochemistry and Microbiology, Rutgers, The State University of New Jersey, New Brunswick, NJ, United States

**Keywords:** mercury, methylmercury, mercuric reductase, organomercury lyase, *merA*, MerB, Hg(II), Thermoprotei

## Abstract

Mercury (Hg) is a highly toxic element due to its high affinity for protein sulfhydryl groups, which upon binding, can destabilize protein structure and decrease enzyme activity. Prokaryotes have evolved enzymatic mechanisms to detoxify inorganic Hg and organic Hg (e.g., MeHg) through the activities of mercuric reductase (MerA) and organomercury lyase (MerB), respectively. Here, the taxonomic distribution and evolution of MerAB was examined in 84,032 archaeal and bacterial genomes, metagenome assembled genomes, and single-cell genomes. Homologs of MerA and MerB were identified in 7.8 and 2.1% percent of genomes, respectively. MerA was identified in the genomes of 10 archaeal and 28 bacterial phyla previously unknown to code for this functionality. Likewise, MerB was identified in 2 archaeal and 11 bacterial phyla previously unknown to encode this functionality. Surprisingly, homologs of MerB were identified in a number of genomes (∼50% of all MerB-encoding genomes) that did not encode MerA, suggesting alternative mechanisms to detoxify Hg(II) once it is generated in the cytoplasm. Phylogenetic reconstruction of MerA place its origin in thermophilic Thermoprotei (Crenarchaeota), consistent with high levels of Hg(II) in geothermal environments, the natural habitat of this archaeal class. MerB appears to have been recruited to the *mer* operon relatively recently and likely among a mesophilic ancestor of Euryarchaeota and Thaumarchaeota. This is consistent with the functional dependence of MerB on MerA and the widespread distribution of mesophilic microorganisms that methylate Hg(II) at lower temperature. Collectively, these results expand the taxonomic and ecological distribution of *mer*-encoded functionalities, and suggest that selection for Hg(II) and MeHg detoxification is dependent not only on the availability and type of mercury compounds in the environment but also the physiological potential of the microbes who inhabit these environments. The expanded diversity and environmental distribution of MerAB identify new targets to prioritize for future research.

## Introduction

Mercury (Hg) is one of the most toxic heavy metals and is a global pollutant emitted by volcanoes, forest fires, and anthropogenic activities such as fossil fuel combustion and smelting and oxidative leaching of pyritic ores (Rooney, 2007; Obrist et al., 2018). The global distribution of Hg poses severe consequences to human and environmental health (Clarkson and Magos, 2006), and the Hg load to environments is only increasing due to increased fossil fuel emissions, changes in land use, and artisanal gold mining (Obrist et al., 2018; Zolnikov and Ramirez Ortiz, 2018). Volatile mercury [Hg(0)] is the primary form of Hg emitted to the atmosphere where it can then be oxidized to Hg(II) and returned to terrestrial and aquatic ecosystems through wet deposition (Selin et al., 2007). Subsequently, Hg(II) can be buried in sediments, reduced to Hg(0) and re-emitted to the atmosphere, or methylated to form organic methylmercury (MeHg) (Selin et al., 2007; Grégoire and Poulain, 2018). MeHg is a highly potent neurotoxin that can enter food webs (Gilmour and Henry, 1991; Watras et al., 1995; Benoit et al., 1998) through uptake by unicellular microbes (Mason et al., 1996; Pickhardt and Fisher, 2007). MeHg can bioaccumulate and its concentration biomagnify in aquatic (Watras et al., 1998; Boyd et al., 2009) and terrestrial (Rimmer et al., 2010) food chains, ultimately impacting the health of animals and plants (Sunderland, 2007; Zhang et al., 2010).

Microorganisms are the primary driver of Hg methylation through the activity of a corrinoid protein, HgcA, and a ferredoxin, HgcB (Gilmour et al., 2013; Parks et al., 2013). Microbial Hg methylation occurs mainly under anaerobic conditions through the activities of iron-reducing bacteria (Fleming et al., 2006), sulfate-reducing bacteria (Compeau and Bartha, 1985; Gilmour et al., 1992), methanogenic archaea (Hamelin et al., 2011; Gilmour et al., 2018), and fermentative bacteria (Parks et al., 2013). Recent bioinformatic investigations of the distribution of *hgcAB* genes in metagenomes (Podar et al., 2015) and in isolate genomes and metagenome assembled genomes (McDaniel et al., 2020) showed that microbial Hg methylation potential is encoded in the genomes of a variety of organisms inhabiting anoxic environments with temperatures < 70°C but was less abundant in environments with temperatures > 70°C and notably absent from human and mammalian microbiomes.

**FIGURE 1:**
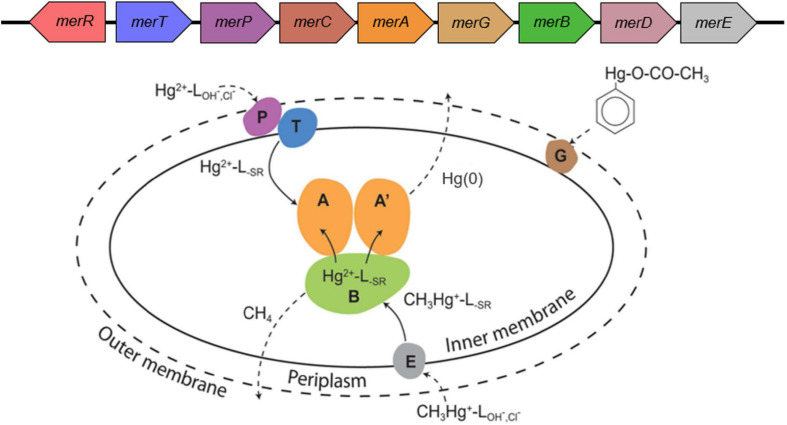
The *mer* detoxification system. **(A)** A generic *mer* operon. **(B)** The cellular *mer*-encoded mercury detoxification mechanisms. The outer cell wall is depicted by a broken line illustrating that not all microbes have an outer membrane; broken line arrows depict diffusion; solid line arrows indicate transport or transformations; L = ligand with subscripts denoting the ligand type. The colors of various Mer proteins correspond with the colors of the genes that encode these proteins in panel **A**. Figure adapted from Lin et al. (2011); Boyd and Barkay (2012).

Functionalities encoded by the *mer* operon are the primary mechanisms microbial cells use for Hg resistance and detoxification (Silver and Hobman, 2007; Lin et al., 2011). At its core, the *mer* operon encodes a homodimeric flavin-dependent disulfide oxidoreductase, termed mercuric reductase (MerA), that functions to reduce Hg(II) to volatile Hg(0) that can then diffuse out of the cell (Boyd and Barkay, 2012; Figure 1). The operon may also code for organomercury lyase (MerB), that catalyzes the protonolytic cleavage of the C-Hg bond in organomercury compounds, among them MeHg (Boyd and Barkay, 2012). This reaction yields a reduced organic moiety, that in the case of MeHg is methane, and Hg(II); the latter is then reduced to Hg(0) through the activity of MerA (Boyd and Barkay, 2012). In addition to MerAB, *mer* operons may code for the periplasmic protein MerP, inner membrane spanning proteins MerT, MerC, MerE, MerF, and MerG that transport Hg(II) to the cytoplasmic MerA (reviewed in Boyd and Barkay, 2012). *mer* operons may also code for transcriptional regulators such as the repressor/activator MerR or anti-activator MerD (reviewed in Barkay et al., 2003; Lin et al., 2011). Through the combined demethylation of MeHg by MerB and decreased concentration of Hg(II) in the cytoplasm by its reduction to gaseous Hg(0) via MerA, the *mer* operon effectively partitions Hg to the gaseous phase, allowing for microbial growth (Barkay et al., 2003; Boyd and Barkay, 2012).

In 2012, a database of publicly available genomes was compiled that included 272 *mer* operons from the genomes of a diverse array of 246 bacterial and archaeal isolates (Boyd and Barkay, 2012). Phylogenetic analyses of MerA homologs compiled in that study, as well as in a previous study (Barkay et al., 2010), found that the earliest branching MerA were from thermophilic bacteria (Aquificales) and that *merA* was acquired in Archaea through lateral gene transfer. Further, it was shown that the complexity of *mer* operons, as indicated by the presence of other *mer* genes found linked to MerA in an operonic structure, increased with evolutionary time through recruitment of additional functionalities. As a result, the efficacy and function of the *mer* detoxification machine was improved including a broadened spectrum of mercurials that could be detoxified by the addition of MerB (Boyd and Barkay, 2012). Targeted studies of the diversity and distribution of MerA-encoding genes have since shown a greater diversity in natural environments than was found in the complete genomes of isolates. This includes diverse *merA* detected in studies of high Arctic snow, freshwater, and sea-ice brine (Møller et al., 2014), in Antarctic sea ice (Gionfriddo et al., 2016), in deep and hot brine environments of the Red Sea (Sayed et al., 2014; Maged et al., 2019), in bioreactors (Shi et al., 2019), and in mercury-contaminated rice paddies in China (Vishnivetskaya et al., 2018), among others. While *merA* is estimated to be roughly ten-times more abundant than *merB* in genomes of isolates (Boyd and Barkay, 2012), more recent metagenomics studies have shown that the number of genomes that encode *merB* is roughly equivalent to those that encode *hgcAB* (Christensen et al., 2019). This is a significant finding considering the global prevalence and distribution of organisms with the ability to generate MeHg (despite it being a relatively rare microbial trait) through the activity of HgcAB (Podar et al., 2015; McDaniel et al., 2020) and the potential for MerB-encoding organisms to balance MeHg production in the environment through demethylation.

Since the original *mer* database was compiled in 2012 (Boyd and Barkay, 2012), significant advances in sequencing technologies, including the broad application of metagenomics and single cell genomics in diverse environmental systems (Bowers et al., 2017; Parks et al., 2017, 2020), have substantively increased the known diversity of microbial life (Brown et al., 2015; Chen L.X. et al., 2018). Yet, the diversity of MerAB protein homologs in these newly discovered microbial genomes remains uncharacterized. In the present study, bioinformatic approaches were applied to publicly available isolate genomes, metagenomic assembled genomes (MAGs), and single cell genomes (SCGs). Genomes, MAGs and SCGs were obtained from the Department of Energy (DOE) Integrated Microbial Genomes/Microbiomes (IMG/M) database (as of May 2020) to investigate the taxonomic distribution and diversity of MerAB, the centerpieces of the *mer* Hg detoxification and MeHg degradation machinery (Boyd and Barkay, 2012). Phylogenetic approaches were applied to reconstruct the evolutionary history of MerA homologs and to provide insight into when MerB was first recruited to the *mer* operon. Finally, potential alternative mechanisms allowing for Hg(II) detoxification in cells that were found to encode MerB but not MerA are proposed to explain how toxic effects of Hg(II) generation from demethylation of MeHg might be overcome.

## Materials and Methods

### Generation of a MerA and MerB Homolog Database

Mercuric reductase (MerA) protein homologs were compiled from publicly available genomes deposited in the DOE IMG/M database (Chen I.M.A. et al., 2018) including isolate genomes, MAGs, and SCGs, in May 2020, using BLASTp within IMG (Markowitz et al., 2006) and MerA from the bacterial transposon Tn*501* [CAA77323.1; Stanisich et al., 1977] and the archaeal taxon *Saccharolobus solfataricus* P2 [AAK42805.1; Schelert et al., 2004] as queries. Additional sequences were identified with the Gene Search function within IMG/M with input query terms “mercury(II) reductase,” “MerA,” and “mercuric reductase.” All putative MerA sequences were aligned with the PROMALS3D structural alignment tool (Pei et al., 2008) using the protein crystal structure of Tn*501* MerA [PDB ID: 1ZK7 (Ledwidge et al., 2005)] as the alignment thread.

Aligned putative MerA were manually examined for the presence of sequence signatures that have been experimentally shown to be essential for MerA activity, as previously described (Barkay et al., 2003; Boyd and Barkay, 2012). Specifically, homologs were screened for the presence of the vicinal cysteine pair at the carboxy terminus (aligned positions 628 and 629; numbering in reference to MerA from *Bacillus cereus* RC607 [BAB62433.1; Wang et al., 1989; Gupta et al., 1999] and for the conserved cysteine pair at positions 207 and 212 in the redox active site, tyrosine at position 264 (Rennex et al., 1993), and tyrosine at position 605 for bacterial MerA and phenylalanine at position 605 for archaeal MerA (Simbahan et al., 2005).

The IMG/M database was also queried for the presence of alkylmercury or organomercurial lyase (MerB) using BLASTp and MerB from the *Escherichia coli* plasmid R831b [AAB49639.1; Pitts and Summers, 2002], *Halobacterium* sp. DL1 [AHG04994.1] and *Candidatus* Nitrososphaera evergladensis SR1 [AIF84719.1; Zhalnina et al., 2014] as queries. Additional MerB homologs were identified with the Gene Search function within IMG/M with input query terms “merB,” “alkylmercury lyase,” “alkylmercury lyase-like,” “organomercury lyase,” and “organomercurial lyase.” Homologs were aligned with the PROMALS3D structural alignment tool specifying the protein crystal structure of MerB from plasmid R831b [PDB ID: 3F0P (Lafrance-Vanasse et al., 2009) as the alignment thread. Aligned MerB homologs were examined for the presence of the following sequence signatures: cysteine at position 96 (numbering in reference to MerB from R831b), aspartic acid at position 99, and cysteines at position 159 and 117 (Pitts and Summers, 2002; Lafrance-Vanasse et al., 2009). Pairwise sequence similarity among MerA or MerB homologs encoded in the same genome were determined using the Sequence Manipulation Suite: Version 2 (Stothard, 2000).

### Phylogenetic Analysis of MerA and MerB

MerA protein homologs were aligned using Clustal Omega version 1.2.4 (Sievers et al., 2011), using default settings with dihydrolipoamide dehydrogenase from *Magnetospirillum magneticum* AMB-1 (YP_423326), *Thermus thermophilus* HB27 (YP_005669), *Pseudomonas fluorescens* Pf0-1 (WP_011336663.1), *Desulfurococcus amylolyticus* DSM 16532 (AFL66567.1), and *Saccharolobus solfataricus* (SAI85240.1) serving as outgroups. N terminal “NmerA” sequences were trimmed from the alignment block as previously described (Barkay et al., 2010; Boyd and Barkay, 2012). The aligned homologs were subjected to Modelfinder included in IQ-TREE vers. 1.6.12 (Kalyaanamoorthy et al., 2017) to identify the optimal substitution model and parameters for the phylogenetic reconstruction of MerA. The phylogeny of MerA was evaluated with IQ-TREE multicore version 1.6.11 (Nguyen et al., 2015; Hoang et al., 2018) with the LG amino acid substitution matrix, empirically counted frequencies from the alignment, a discrete four category gamma substitution model (gamma shape parameter = 0.9583), and a defined proportion of invariant sites of 0.0080, specifying 1000 ultrafast bootstraps. The phylogenetic tree was visualized in ITOL (Letunic and Bork, 2019). The MerA phylogenetic tree is available in newick format in Supplementary Dataset 1. All aligned MerA homolog sequences used for the construction of the phylogenetic tree, along with the plasmid MerA homologs not used for the phylogenetic tree construction, are available in fasta format in Supplementary Dataset 2.

Compiled MerB protein homologs were aligned with Clustal Omega version 1.2.4 (Sievers et al., 2011), specifying default settings. The aligned homologs were subjected to Modelfinder included in IQ-TREE version 1.6.12 as mentioned above. The phylogeny of MerB was evaluated with IQ-TREE multicore version 1.6.11 as mentioned above but with gamma shape parameter = 1.6454, defined proportion of invariant sites of 0.0000, and 1000 ultrafast bootstraps. The phylogenetic tree was visualized in ITOL. The MerB phylogenetic tree is available in newick format in Supplementary Dataset 3. All aligned MerB homolog sequences are available in fasta format in Supplementary Dataset 4.

## Results and Discussion

### Overview of the Genomic Database Queried for MerA and MerB

A total of 84,032 isolate genomes, MAGs, and SCGs (heretofore referred to as genomes) available in the IMG/M database as of May 2020 were queried for homologs of MerA and MerB. Of the 84,032 genomes queried, 80,925 were bacterial, 1959 were archaeal, and 1148 were plasmid genomes. Furthermore, 70,633 were isolate genomes (including plasmid genomes), 9590 were MAGs, and 3809 were SCGs. MerA and/or MerB homologs were identified in 7161 isolate genomes, 480 MAGs and 76 SCGs (Supplementary Table 1).

### Taxonomic Distribution of MerA Encoding Genes

A total of 7544 MerA homologs were identified (Supplementary Table 1) and these were distributed among 6574 archaeal and bacterial genomes (Table 1; Supplementary Table 1), with 813 of these genomes encoding for multiple (up to 8) homologs (Table 2). The 7544 MerA homologs were derived from 6063 isolate and plasmid genomes, 446 MAGs, and 65 SCGs (Supplementary Table 1).

**TABLE 1:** Number of genomes that encode MerA or MerB protein homologs and their distribution at the Phylum level in the database constructed in 2012 (Boyd and Barkay, 2012) and here in 2020.

	**Number of genomes encoding MerA homologs**		Number of genomes encoding MerB homologs*	
**Phylum/Class**	**2020**	**2012**	**2020**	**2012**
**Archaea**	**271**	**24**	**11**	**−**

*Candidatus* Bathyarchaeota	1	−	−	−
*Candidatus* Diapherotrites	1	−	−	−
*Candidatus* Geoarchaeota	4	−	−	−
*Candidatus* Geothermarchaeota	2	−	−	−
*Candidatus* Heimdallarchaeota	1	−	3	−
*Candidatus* Micrarchaeota	22	−	−	−
*Candidatus* Odinarchaeota	1	−	−	−
*Candidatus* Parvarchaeota	10	−	−	−
Crenarchaeota	139	15	−	−
Euryarchaeota	65	9	8	−
Nanoarchaeota	1	−	−	−
Thaumarchaeota	10	−	−	−
unclassified	14	−	−	−

**Bacteria**	**6274**	**253**	**1778**	**40**

Acidobacteria	7	−	−	−
Actinobacteria	958	36	468	13
Aquificae	7	3	−	−
Armatimonadetes	1	−	−	−
Bacteroidetes	131	5	3	4
Balneolaeota	4	−	−	−
Candidate division GAL15	1	−	−	−
Candidate division NC10	−	−	1	−
Candidate division Zixibacteria	−	−	1	−
*Candidatus* Atribacteria	1	−	−	−
*Candidatus* Azambacteria	1	−	−	−
*Candidatus* Bipolaricaulota	1	−	−	−
*Candidatus* Blackburnbacteria	1	−	−	−
*Candidatus* Daviesbacteria	7	−	−	−
*Candidatus* Giovannonibacteria	10	−	−	−
*Candidatus* Gottesmanbacteria	1	−	−	−
*Candidatus* Kaiserbacteria	1	−	−	−
*Candidatus* Kryptonia	1	−	−	−
*Candidatus* Microgenomates	2	−	−	−
*Candidatus* Pacebacteria	1	−	−	−
*Candidatus* Parcubacteria	2	−	−	−
*Candidatus* Peregrinibacteria	5	−	−	−
*Candidatus* Roizmanbacteria	2	−	−	−
*Candidatus* Rokubacteria	2	−	1	−
*Candidatus* Sumerlaeota	1	−	−	−
Chlamydiae	1	−	1	−
Chloroflexi	32	1	7	−
Cyanobacteria	1	−	−	−
Deferribacteres	2	−	−	−
Deinococcus-Thermus	14	2	2	−
Firmicutes	960	42	797	5
Fusobacteria	−	−	1	−
Gemmatimonadetes	−	−	1	−
Ignavibacteriae	15	−	−	−
Nitrospirae	18	1	6	−
Planctomycetes	1	−	−	−
Proteobacteria	4050	161	478	18
Alphaproteobacteria	668	25	84	3
Betaproteobacteria	412	24	72	2
Gammaproteobacteria	2869	104	298	13
Deltaproteobacteria	37	8	10	−
Epsilonproteobacteria	1	−	−	−
Zetaproteobacteria	27	−	4	−
Acidithiobacillia	25	−	6	−
Hydrogenophilalia	2	−	1	−
Oligoflexia	6	−	2	−
*Candidatus* Muproteobacteria	1	−	−	−
Unclassified	2	−	1	−
Rhodothermaeota	1	−	−	−
Spirochaetes	3	−	5	−
Synergistetes	−	−	3	−
Tenericutes	11	1	−	−
Verrucomicrobia	7	1	−	−
unclassified	10	−	3	−

**Plasmid:Bacteria**	**29**	**−**	**6**	**−**

Alphaproteobacteria	1	−	1	−
Betaproteobacteria	1	−		−
Gammaproteobacteria	25	−	4	−
Unclassified Proteobacteria	2	−	1	−
Ynclassified	−	3	−	3

*In 2012 surveys, MerB was only noted when linked to a MerA that were identified by homology searches of the IMG database. In 2020, independent searches for MerB were performed. In the case of Proteobacteria and plasmids, taxonomic assignment at the class level is also shown.

**TABLE 2:** Abundance of archaeal, bacterial, and plasmid genomes that encode one or more MerA or MerB protein homologs.

**MerA copies per genome**	**1**	**2**	**3**	**4**	**5**	**6**	**8**	**Total**
Archaea	270	−	1	−	−	−	−	271
Bacteria	5497	668	74	29	2	3	1	6274
Plasmid:Bacteria	28	1	−	−	−	−	−	29

**MerB copies per genome**	**1**	**2**	**3**	**4**	**8**	**Total**		

Archaea	11	−	−	−	−	11		
Bacteria	1685	64	15	13	1	1778		
Plasmid:Bacteria	5	1	−	−	−	6		

Among the 1959 archaeal genomes examined, a total of 271 (13.8%) encoded MerA homologs and these were distributed among 12 phyla (Table 1). The 307 archaeal MerA protein homologs in the current database represents a roughly ten-fold increase over the number identified in the 2012 database (Table 1; Boyd and Barkay, 2012), and include homologs from an additional 10 phyla and candidate phyla. These include genomes affiliated with the Thaumarchaeota, Nanoarchaeota, and various candidate phyla including *Candidatus* Micrarchaeota, *Candidatus* Parvarchaeota, and *Candidatus* Geoarchaeota. While this is an expansion of our previous inventories, the added archaeal genomes are from taxa that generally inhabit hot springs, in particular acidic hot springs that are acidified through microbially-mediated O_2_-dependent oxidation of sulfur compounds (Colman et al., 2018). The presence of MerA encoding genomes from taxa in these environments is thus consistent with the aerobic conditions that have been previously suggested to enhance Hg toxicity (Barkay et al., 2010). Nearly all (99.6% of total) MerA encoding archaeal genomes coded for a single copy (Table 2) with the exception of *Cuniculiplasma divulgatum* S5 (Euryarchaeota; Diaforarchaea group) that encodes 3 copies. Two of the three copies of MerA encoded in *C. divulgatum* are highly similar (89.2% sequence identities), suggesting a possible gene amplification within this lineage. *C. divulgatum* S5 is an acidophilic, mesophilic, facultatively anaerobic heterotroph isolated from a sulfide ore mine (Golyshina et al., 2016). Sulfide ores often have trace Hg(II) (Monecke et al., 2016; Fallon et al., 2017) that, when liberated through oxidative weathering, might be expected to select for duplicons encoding for MerA to increase Hg(II) detoxification capabilities through a gene dosage effect (Kondrashov, 2012).

Among the 84,032 bacterial genomes examined, a total of 6274 (7.75%) encoded MerA homologs (Table 1). An additional 29 proteobacterial plasmid genomes encoded MerA homologs (Table 1), out of the 1148 plasmid genomes queried. The 7207 MerA homologs were distributed among 37 phyla, candidate phyla, or candidate divisions. The number of genomes that encode MerA in the present genomic database is roughly 25-fold greater than those identified in 2012 (Table 1; Boyd and Barkay, 2012) and includes MerA from 26 new bacterial phyla including Acidobacteria, Balneolaeota, and *Candidatus* Giovannonibacteria, among others (Table 1). Further, homologs of MerA were identified among several new proteobacterial classes such as the Epsilonproteobacteria (e.g., *Campylobacter concisus*), the iron-oxidizing Zetaproteobacteria, and the thermophilic Hydrogenophilalia. Of the 6274 bacterial genomes that encode MerA, 777 encode multiple copies (Table 2), including one genome (*Cupriavidus metallidurans* H1130) that encodes 8 copies. While *C. metallidurans* strain H1130 was isolated from the blood of a patient suffering from nosocomial septicemia (Monsieurs et al., 2013), other *C. metallidurans* strains are frequently isolated from environments impacted by mining, metallurgic, and chemical industries (Diels and Mergeay, 1990; Goris et al., 2001). All 8 copies of MerA in this genome are identical (100% sequence identities) suggesting recent duplication events within this lineage, perhaps a response to increase detoxification efficiency due to Hg(II) exposure.

Several MerA homologs contained residues that vary from characterized sequences, in particular, at position 605 (numbering in reference to MerA of *Bacillus cereus* RC607). Variants at this position were also detected in a previous characterization of MerA diversity (Boyd and Barkay, 2012), with tyrosine identified in bacterial MerA homologs and phenylalanine in archaeal homologs (Supplementary Figure 1). We detected 46 bacterial MerA homologs that feature a phenylalanine instead of a tyrosine at position 605 and 2 archaeal MerA homologs that feature a tyrosine instead of a phenylalanine in this same position (Supplementary Table 2). The 46 bacterial MerA variants are distributed among 15 phyla, while the 2 archaeal variants both belong to members of the Euryarchaeota (Supplementary Table 2). All but two of these MerA variants are the sole MerA homologs in their respective genomes, while 2 of these variants co-exist in the genome with a MerA homolog with the typical residue at that position (canonical MerA) (Supplementary Table 1). To date, no MerA with a noncanonical substitution in position 605 has been tested for activity. Demonstrating activity of such variants may lead to a better understanding of the role of this residue in Hg(II) reduction by MerA.

### Taxonomic Distribution of MerB Encoding Genes

A total of 1936 alkylmercury lyase (MerB) homologs were identified in publicly available genomes of the IMG database. These 1936 MerB protein homologs were distributed among 1722 genomes, 61 MAGs, and 12 SCGs (Supplementary Table 1), with 94 genomes encoding multiple homologs (up to 8; Table 2). While several genomes analyzed herein were shown to encode multiple copies of MerB, to date no genome has been shown to encode multiple copies of HgcAB (Podar et al., 2015; McDaniel et al., 2020).

Among the 1959 archaeal genomes examined, 11 (0.6%) encoded MerB homologs and these were distributed across 2 phyla: *Candidatus* Heimdallarchaeota and Euryarchaeota (Table 1). Demethylation of MeHg has not yet been described among the Archaea and a previous study of available isolate genomes in 2012 did not identify MerB homologs among the MerA-linked loci in archaeal genomes (Boyd and Barkay, 2012). The detection of MerB homologs in archaeal genomes is consistent with the significant expansion of the taxonomic diversity of the *mer* operons in the present study. All archaeal MerB-encoding genomes encoded for a single MerB copy (Table 2). Interestingly, MerB homologs were not identified among genomes of members of the Crenarchaeota, the phylum that featured the largest number of MerA homologs (Table 1).

Among the 80,925 bacterial genomes examined, 1778 (2.4%) encoded MerB protein homologs and these were distributed across 15 different phyla, candidate phyla, and divisions (Table 1), including 11 new phyla and candidate divisions not reported to encode MerB in the 2012 genomic database (Boyd and Barkay, 2012). These include members of the Nitrospirae, Spirochetes, and Chloroflexi along with the proteobacterial classes Deltaproteobacteria, Zetaproteobacteria, and Acidithiobacillia, among others (Table 1). Additionally, 7 homologs were identified on 6 proteobacterial plasmid genomes, with 1 plasmid containing 2 homologs (Table 2). Studies on organomercury resistance and degradation of MeHg have largely been focused on proteobacterial (Pitts and Summers, 2002) and bacilli (Matsui et al., 2016) strains. The broad taxon distribution of MerB homologs reported here calls for an examination of enzyme activity in more organisms where this activity has not been tested before.

Among the 1795 MerB-encoding genomes, 165 encoded multiple copies, up to 8 in the genome of *Bacillus cereus* strain #17 (Supplementary Table 3; Table 2). This strain was isolated from a mouse gut microbiome sample (Böhm et al., 2015). These 8 homologs probably originated from 2 different ancestral genes, with each of these genes undergoing multiple (quadruple) duplication events and thus forming 2 groups that each comprise 4 identical (100% sequence identities) homologs; the 100% similarity within each group suggests that the duplication events took place recently. This strain further encodes one additional group of 4 identical copies of the MerB-like variant, 99Ser, which is described in detail below. *Bacillus* strains have been previously found to encode multiple *merB* genes, designated as *merB1, merB2, merB3* (Gupta et al., 1999; Huang et al., 1999; Chien et al., 2010; Boyd and Barkay, 2012). Heterologous expression and biochemical characterization reveal *Bacillus* MerB1 to preferentially degrade alkylmercurials (methylmercury, ethylmercury, thimerosal, and *p*-chloromercuribenzoate) whereas *Bacillus* MerB3 preferentially degrades arylmercurials (*p*-chloromercuribenzoate) (Chien et al., 2010). It is likely that the multiple copies of MerB in the *B. cereus* strain #17 broadens the range of organomercurials that this strain can detoxifiy. The *B. cereus* #17 genome also contains 4 copies of *merA*. While little information is available on the mouse host from which this strain was isolated (Böhm et al., 2015), it is possible that the diet of this mouse was rich in organomercurials, leading to selection for a strain that encoded such a diverse array of MerAB. Alternatively, it is possible that this strain or a recent ancestor of this strain was exposed to environmental organomercurials that selected for this high level of redundant functionalities prior to its recruitment as a member of the mouse gut microbiome.

MerB protein homologs were initially identified and screened using specific amino acid signatures, as previously described (Pitts and Summers, 2002; Lafrance-Vanasse et al., 2009). This includes cysteine at position 96 (numbering in reference to MerB from plasmid R831b), aspartic acid at position 99, and cysteines at position 159 and 117. Apart from the MerB homologs identified, an additional number of MerB-like homologs were identified which differed in these sequence signatures. When a homolog differed in at least one of the aforementioned amino acid signatures, these homologs were designated as MerB-like variants 99Ser and 117alt (Supplementary Figure 1). In MerB-like variant 99Ser, serine is found at position 99 instead of aspartic acid, and in MerB-like variant 117alt, an alternative amino acid is present at position 117 instead of cysteine (Supplementary Figure 1; Supplementary Table 3). The 13 MerB-like 99Ser variants were identified in genomes that all contain at least one MerB with the aforementioned canonical sequence signatures; these genomes belonged to members of the bacterial phylum Firmicutes. Previous structural and biochemical characterization of representatives of the 99Ser MerB-like variants (Wahba et al., 2016) has shown that they have expanded range of affinities for metals relative to canonical MerB. Specifically, this variant has an affinity to bind Cu(II) that is displaced by Hg(II) when present. Hg(II) is a product of MerB-dependent demethylation of MeHg, and MerB binds Hg(II) so that it cannot diffuse into the cytoplasm; MerA then extracts the metal for reduction (Wahba et al., 2016). The physiological role of these variant enzyme with an affinity for other divalent metals is thus not well understood especially when a native MerB 99Ser, in *B. megaterium* MerB2, does not bind Cu(II) (Wahba et al., 2016). However, it is possible that they function as more generalized organic metalloid detoxification enzymes than canonical MerB. If so, strains carrying MerB-like 99Ser in addition to the canonical MerB may possess an expanded range of metal resistance.

The importance of the 117Cys to MerB function is not clear. Pitts and Summers (2002), who initially identified it as a conserved amino acid, attributed a structural rather than a catalytic role to this moiety (Barkay et al., 2003). Current models of MerB show 117Cys to be deeply buried in the hydrophobic core of the protein and removed from solvent, and thus interactions with putative substrates (Di Lello et al., 2004). Of the total 563 MerB-like 117alt variant homologs, 20 were identified in genomes with a canonical MerB and these genomes belong to Actinobacteria and Firmicutes (Supplementary Table 3). The remainder of the 563 MerB 117alt variants were the only MerB homologs detected in their corresponding genomes and they are distributed in Actinobacteria, Bacteroidetes, Chloroflexi, Deinococcus-Thermus, Firmicutes, Proteobacteria, and Spirochetes (Supplementary Table 3). Crystallographic, structural, and biochemical studies of MerB have focused on the essential role of Cys96, Asp99, and Cys159 residues (Lafrance-Vanasse et al., 2009; Wahba et al., 2016; Roy et al., 2020), likely due to their strict conservation and catalytic role. To the best of our knowledge, the activity and/or substrate range of variants with substitution at position 117 has not been tested; a C117S mutant was insoluble and could not be tested (Pitts and Summers, 2002). The widespread taxonomic distribution of this variant in genomes lacking a canonical MerB suggests that the activity of 117alt should be tested for a better understanding of the physiological and ecological role of this enzyme.

### Co-distribution of MerA and MerB Homologs in Genomes

**FIGURE 2:**
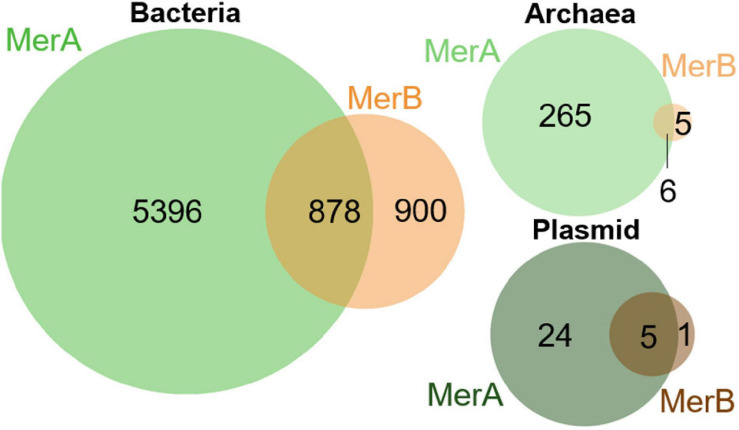
Venn diagrams reporting the number of genomes with MerA and/or MerB protein homologs. Analyzed genomes included those from Bacteria, Archaea, and plasmids that encode only MerA homologs, only MerB homologs, or that encode both MerAB. The number of genomes in each category is indicated.

A total of 5685 genomes encoded *mer* operons that included MerA but not MerB. This suggests a decreased spectrum of Hg compounds that these cells can detoxify. Interestingly, a total of 906 genomes, slightly higher than 50% of all *merB*-carrying genomes (a total of 1795 genomes), encoded MerB protein homologs but no homolog of MerA (Figure 2; Supplementary Table 1). These included 10 draft genomes and 896 finished genomes indicating that this observation is unlikely to be an artifact of incomplete genome sequencing. Moreover, of the 906 MerB-encoding genomes that lacked a MerA homolog, 900 belonged to Bacteria, 1 was carried by a bacterial plasmid, and 5 belonged to Archaea. Of these 906 genomes, 870 are isolate genomes, 29 are MAGs, and 7 are SCGs. It should be noted that, in the case of MAGs, it is possible that a MerA-encoding plasmid could have been missed during the assembly process (Maguire et al., 2020). The presence of a plasmid-encoded MerA could be identified in future studies through application of specific algorithms that are designed to identify plasmid genomes in assembled metagenomic data (Rozov et al., 2017; Antipov et al., 2019). Of the 6574 MerA-encoding genomes, 249 encoded only 117alt MerB-like variants, 36 encoded 117alt MerB-like variants along with at least one canonical MerB, and 9 genomes encoded a MerB-like 99Ser variant along with a canonical MerB. Of the 895 genomes that encode a MerB homolog but lacked a MerA homolog, 732 (81.3%) belong to the genera *Clostridioides*, *Mycobacteroides*, *Clostridium*, *Staphylococcus*, *Corynebacterium*, *Streptomyces*, *Paeniclostridium*, *Amycolatopsis*, *Thioalkalivibrio*, *Mycobacterium*, *Nocardia*, or *Enterobacter* (Supplementary Table 1). These genera include aerobic, facultatively anaerobic, microaerophilic, and anaerobic representatives, indicating that the ability to demethylate MeHg via MerB without a dedicated strategy to reduce and volatilize Hg(II) via MerA is not necessarily related to the strains ability to use oxygen. Additionally, none of the genomes that encode only MerB code for homologs of HgcAB. Further, mapping of the presence of MerA on an unrooted MerB and MerB-like phylogeny failed to reveal a pattern in the distribution of organismal genomes that encode only MerB homologs versus those that encode both MerAB (Supplementary Figure 1). To the best of the authors’ knowledge, activities of organisms carrying only MerB or organisms that encode only MerB have yet to be reported (Boyd and Barkay, 2012; Grégoire and Poulain, 2018). Organomercury degradation without removal of Hg(II) may present challenges to the activity of the enzyme and the growth and survival of the organism. First, current models suggest a direct transfer of the Cys96-bound Hg(II) from MerB to MerA (Benison et al., 2004) raising questions about how MerB catalysis can continue in absence of MerA. Second, production of intracellular Hg(II) from MeHg by MerB, without the presence of Hg(II) reductive capability in the form of MerA, would be expected to be physiologically detrimental to cells due to an increase in reactive oxygen species as a result of a depletion of cellular redox buffers and increased enzyme destabilization by Hg(II) (Miller et al., 1991; Ariza and Williams, 1999; Valko et al., 2005). The observation of numerous genomes that encode MerB but not MerA and the observation that MerB in absence of MerR results in hypersensitivity to organomercury (Kiyono et al., 1995) suggests that cells must have evolved alternative mechanisms of mitigating the toxic effects of Hg(II) produced from MeHg demethylation. Here, potential alternative mechanisms of Hg(II) reduction in cells are examined with the goal of identifying putative functionalities that could mitigate the toxicity of Hg(II) generated by MerB in strains that lack MerA (Table 3). The suggested mechanisms could be tested for their role in organomercury detoxification to complement existing paradigms on microbial transformations in mercury biogeochemistry.

**TABLE 3:** Possible Hg(II) detoxification mechanisms independent of the *mer* system^1^.

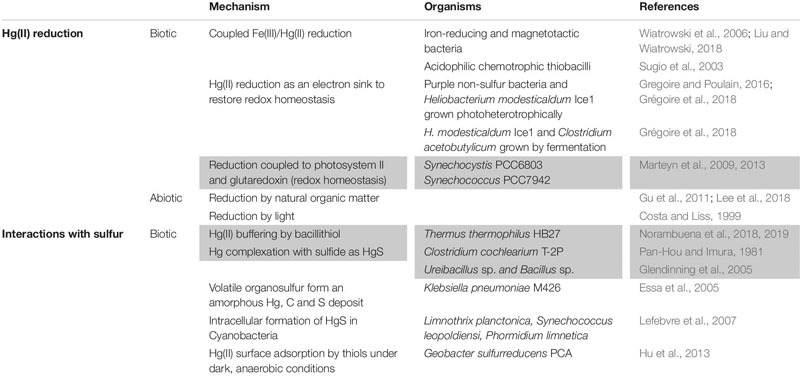

*^1^Cells highlighted in gray indicate the documented role for the listed mechanism in resistance to Hg(II).*

Several non-*mer* Hg(II) reduction mechanisms have been described [for a comprehensive list of such mechanisms see Grégoire and Poulain (2018)] and these, if coupled to organomercury degradation by MerB, could result in detoxification (Table 3). Constitutive Hg(II) reduction was observed in the dissimilatory metal-reducing bacteria *Shewanella oneidensis* MR-1, *Geobacter sulfurreducens* PCA, and *Geobacter metallireducens* GS-15 (Wiatrowski et al., 2006). Strain MR-1 does not encode MerA and while *Geobacter* do encode divergent MerA homologs, their direct role in Hg(II) reduction is not clear (Hu et al., 2013). For *S. oneidensis* MR-1 cells but not *G. sulfurreducens* PCA or *G. metallireducens* GS-15 cells, Hg(II) reduction was detected when they were provided with oxygen or fumarate as electron acceptors. However, all three strains were able to reduce Hg(II) when ferric oxyhydroxide was provided as a terminal electron acceptor (Wiatrowski et al., 2006). Subsequently, it was shown that the product of ferric oxyhydroxide reduction, magnetite, is likely responsible for the reduction of Hg(II) in these cells (Wiatrowski et al., 2009). This conclusion was further supported when Hg(II) was shown to be reduced by biogenic magnetite in the membrane of the magnetosome of two magnetotactic bacteria (*Magnetospirillum gryphiswaldense* MSR-1 and *Magnetospirillum magnetotacticum* MS-1) (Liu and Wiatrowski, 2018). Thus, Hg(II) reduction in these cells likely occurs via coupled iron and Hg(II) redox reactions as an alternative mechanism of Hg(II) reduction. This mechanism is therefore a slight variation on the reported reduction of Hg(II) by chemotrophic and mercury-resistant acidophilic ferrous iron-oxidizing thiobacilli (Sugio et al., 2003). In these cells, Hg(II), instead of oxygen, is reduced at the terminus of an electron transport chain that is initiated by the oxidation of ferrous iron.

Hg(II) can also be reduced by cellular processes that result in the production of excess reducing power (Table 3). For example, Hg(II) was reduced in cultures of purple non-sulfur bacteria (*Rhodobacter capsulatus*, *Rhodobacter sphaeroides*, and *Rhodopseudomonas palustris*) grown photoheterotrophically (Gregoire and Poulain, 2016). Using light as an energy source and acetate or butyrate as carbon source, these purple non-sulfur bacteria grow better in presence of sublethal concentrations of Hg(II) than without it. The authors elegantly show that by serving as an additional electron sink, Hg(II) can restore redox homeostasis thereby enhancing the overall metabolism of the cells (Gregoire and Poulain, 2016). Similarly, in cultures of obligate anaerobes from the order Clostridiales (*Clostridium acetobutylicum* ATCC 824 and *Heliobacterium modesticaldum* Ice1), MerA-independent Hg(II) reduction was observed during fermentation of pyruvate, presumably due to the production of reduced redox cofactors such as low potential ferredoxin (Grégoire et al., 2018). Similarly, *H. modesticaldum* Ice1 was shown to reduce Hg(II) when grown photoheterotrophically with pyruvate as a carbon source. In these cases, it is possible that low potential ferredoxins produced during phototrophic or organotrophic metabolism are responsible for Hg(II) reduction (Grégoire et al., 2018). In the examples outlined above, Hg(II) is reduced due to its electrophilic properties, leading to the production of volatile Hg(0).

Previous bioinformatics studies have shown that oxygenic Cyanobacteria do not encode MerA (Barkay et al., 2010), and the expanded MerA database reported here supports this conclusion with the exception of a single MerA-encoding *Halothece* genome (IMG ID: 2795385387) recovered from a halite pinnacle. Despite not encoding MerA, the cyanobacterium *Synechocystis* PCC6803 was shown to reduce Hg(II), likely mediated by a redox homeostasis system powered by low potential ferredoxins generated by photosystem II, like those mentioned above (Marteyn et al., 2009). Subsequently, a MerA-like enzyme was identified that was dependent on interactions with glutaredoxin for its activity; that activity resulted in the reduction and increased tolerance to Hg(II) as well as to uranyl acetate (Marteyn et al., 2013). While such mechanisms may help explain light-dependent Hg(II) reduction by phototrophs (Ben-Bassat and Mayer, 1978; Mason et al., 1995), none of the genomes that encode MerB and that lack homologs of MerA were derived from Cyanobacteria (Supplementary Table 1).

Finally, an array of studies have documented Hg(II) reduction by abiotic mechanisms that themselves are often indirectly connected to specific microbial activities and associated metabolites (Table 3). Dissolved organic matter (DOM) is an enormous effector of mercury bioavailability and this is largely attributable to interactions with organo-thiols (RSHg^+^) and Hg(II) (Ravichandran, 2004). Likewise, Hg(II) has been shown to be reduced by humic acids associated with natural organic matter (NOM) in aquatic environments (Gu et al., 2011). Reduction of Hg(II) by humic acids appears to be common in anoxic environments with abundant NOM explaining numerous observations of *mer*-independent formation of Hg(0) in such environments (*see* Grégoire et al., 2018). Interestingly, intricate interactions between microbes and NOM have been suggested to impact Hg(II) reduction. For example, NOM-enhanced Hg(II) reduction by *S. oneidensis* MR-1 was shown to occur by diverting electrons generated during anaerobic respiration to Hg(II) via NOM (Lee et al., 2018). Further, inhibition of Hg(II) reduction was observed at higher concentrations of NOM, likely due to sequestration of Hg(II) by RSHg^+^ groups in the NOM. Additionally, reduction of Hg(II) in the presence of DOC has been observed under sunlight in many different types of natural waters (Costa and Liss, 1999). This activity is affected by many factors such the chemistry and molecular weight of the DOC pool, the availability and wavelength of light, pH, dissolved oxygen, and the composition and abundance of cations and anions (Luo et al., 2020). Photoreduction of Hg(II) by intracellular algal components has also been documented (Kritee et al., 2018); however, the relevance of this process in mitigating the intracellular toxicity of Hg(II) produced from MeHg remains to be determined in species whose genomes encode MerB but not MerA.

Not surprisingly, several known alternative Hg resistance mechanisms are based on the interactions of Hg(II) with reduced sulfur (Table 3). Furthermore, uptake and transformations of Hg(II) in Hg(II)-methylating or reducing bacteria have been shown to be facilitated by thiols (-SH) and RSHg^+^ on proteins (Yu and Fein, 2017; Thomas et al., 2018, 2019; Song et al., 2020). Such interactions drive Hg behavior at all levels from the intracellular molecular level (Barkay et al., 2003) to the ecosystem level (Ravichandran, 2004) and they often lead to increased Hg(II) resistance. For example, the well characterized *mer* operon in the thermophilic bacterium *Thermus thermophilus* carries a gene, *oah*2, that codes for the synthesis of the low molecular weight thiol bacillithiol (Norambuena et al., 2018, 2019). Deletion mutants of *oah2* as well as those of several genes involved in the reduction of bacillithiol were as sensitive to Hg(II) as a MerA deletion mutant. Furthermore, it was shown that bacillithiol can be oxidized by Hg(II). The authors suggested that along with Hg(II) reduction by MerA, bacillithiol buffers the cell against Hg(II) toxicity (Norambuena et al., 2018, 2019). In another example, the genes encoding capabilities that allow the obligate anaerobe *Clostridium cochleariumm* T-2P to demethylate MeHg and to generate hydrogen sulfide were encoded on a plasmid (Pan-Hou and Imura, 1981). Curing the strain of the plasmid resulted in a loss of both activities and this strain was more sensitive to mercurials than the wild type (Pan-Hou and Imura, 1981). This suggests that MeHg demethylation, likely mediated by MerB, is coupled to H_2_S generation and this results in the formation of insoluble mercury sulfide (HgS; cinnabar). A third example was described in two moderately thermophilic *Bacillus* and one *Ureibacillus* strains that were exposed to Hg(II) which resulted in the formation of a black precipitate, subsequently shown to be HgS by energy dispersive X-ray diffraction analysis (Glendinning et al., 2005). Volatile sulfide was not detected in the growth medium of these cells and it was therefore suggested that enhanced Hg tolerance in these cells is based on the production of non-volatile intracellular thiols that promote sequestration of Hg(II) as HgS (Glendinning et al., 2005). A better understanding of the role of thiols in Hg(II) sequestration in taxa whose genomes encode MerB but not MerA and targeted experiments where strains defective in thiol production are used as controls are needed to determine their potential role in mitigating the toxicity of Hg(II) generated by MeHg demethylation.

Many studies have documented additional interactions of Hg(II) with sulfur (Ravichandran, 2004), that might be considered for their role in Hg(II) resistance. For example, HgS complexes have been documented in *Klebsiella pneumoniae* M426 (Essa et al., 2002). This strain was shown to produce volatile organosulfur products that led to the formation of amorphous precipitates that contained Hg, carbon, and sulfur. Likewise, in some Cyanobacteria, intracellular deposits of HgS were observed and these were suggested to be formed via the precipitation of Hg(II) by intracellular thiol groups (Lefebvre et al., 2007).

The above mechanisms provide a framework for rationalizing how organisms that encode MerB, but not MerA, could demethylate Hg and mitigate the toxic consequences of intracellularly produced Hg(II). The mechanisms described are found in diverse taxa operating a variety of energy metabolisms ranging from aerobic to anaerobic and employing fermentative, heterotrophic, and phototrophic/photosynthetic metabolisms, indicating that MerA-independent Hg(II) detoxification can take place in a variety of ecological contexts. These mechanisms are possibly widespread in biology considering that thiols are universal components of all cells (Rooney, 2007; Benedikter et al., 2018), sulfide is produced in many cells through dissimilatory metabolism [e.g., sulfate or sulfur reduction (Plugge et al., 2011) and assimilatory metabolism; e.g., biosynthesis of cysteine (Kitabatake et al., 2000; Mukai et al., 2017)], and iron metabolism is common place among anaerobic or facultatively anaerobic cells (Weber et al., 2006). Further, reduced cofactors such as low potential ferredoxins are ubiquitous in anaerobic cells, including a variety of phototrophs and chemoautotrophs, the latter of which generate this low potential reductant through flavin-based electron bifurcation or ion-coupled translocation mechanisms (Buckel and Thauer, 2018; Boyd et al., 2020). Such low potential ferredoxins can also be generated through heterotrophic metabolisms, including those that respire, ferment, or photo-assimilate organic carbon (Gregoire and Poulain, 2016; Poudel et al., 2018). The fact that Hg(II) detoxification can be accomplished through these means, when combined with data presented herein revealing the diversity of taxa that encode MerA, indicates that MerA is a superior Hg(II) reducing catalyst with a dedicated role to reduce and thus detoxify Hg(II). The majority of the MerA-independent mechanisms of detoxifying Hg(II), as described above (Table 3), are potentially important to species inhabiting anoxic habitats. Consequently, the distribution of these mechanisms largely among anaerobes is consistent with the emergence of MerA among aerobes, which has been suggested to have taken place only after O_2_ began to accumulate in the biosphere and when HgS minerals were oxidatively weathered thereby increasing the release of toxic Hg(II) (Barkay et al., 2010; Boyd and Barkay, 2012).

### Phylogenetic Reconstruction of MerA Homologs

**FIGURE 3:**
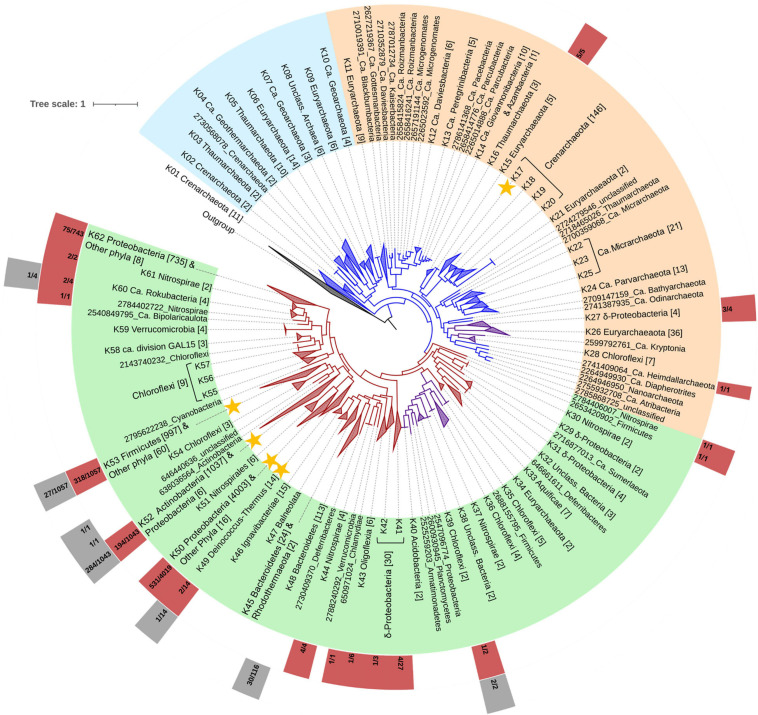
Phylogenetic tree of MerA protein homologs (plasmid MerA not included). Archaeal and bacterial clades are denoted with blue and red color, respectively, while purple-colored clades denote mixed lineages. Colors covering labels of lineages denote the three primary groups of MerA: Group A (light blue), Group B (light orange) and Group C (light green). The outer boxes right to the labels show the presence of MerB (light red box) and MerB-like (gray boxes) homologs in the genomes from which the MerA homologs were derived. Numbers in the light red and gray boxes denote how many of the MerA-encoding genomes within each collapsed node also encoded MerB or MerB-like homologs, respectively. The outgroup consists of 6 bacterial and archaeal dihydrolipoamide dehydrogenase sequences. Tree scale is in amino acid substitutions per site. Yellow stars denote lineages with representatives which their MerA activity has been confirmed. Single lineages not grouped with other clades are depicted with their gene ID and phylum assignment.

The discovery of previously unidentified and diverse MerA protein homologs motivated a phylogenetic analysis to further evaluate the evolutionary history of MerA-mediated Hg(II) reduction (Figure 3). In the presented phylogenetic tree, MerA lineages were collapsed at the phylum level or at the class level in the case of Proteobacteria. Lineages were not collapsed when a monophyletic clade could not be forced at the taxonomic ranks outlined above or when MerA belonged to multiple phyla within a lineage. When a lineage was collapsed, it was additionally assigned a numeric designation (e.g., K02 Crenarchaeota; Figure 3; Supplementary Table 4) and the number of MerA homologs in each lineage is depicted in brackets. Non-collapsed clades were not provided with a “K” designation. Rapid bootstraps values were also calculated and are presented in Supplementary Dataset 1. Plasmid encoded MerA protein homologs were excluded from the analysis due to their polyphyletic nature (Hülter et al., 2017).

Phylogenetic reconstruction of MerA proteins showed that lineages broadly correspond to the phylum taxonomic level of the genome from which they originate, similar to prior analyses of MerA phylogeny (Barkay et al., 2010; Boyd and Barkay, 2012). The MerA phylogeny consists of three large empirically defined groups of sequences: Group A that consists exclusively of MerA from archaeal lineages, Group B that consists primarily of MerA from archaeal lineages along with several recently described bacterial lineages (i.e., Kryptonia, *Candidatus* Giovannonibacteria, *Candidatus* Peregrinibacteria among others), δ-Proteobacteria, and Chloroflexi, and Group C that consists primarily of MerA from bacterial lineages as well as MerA from a Euryarchaeota lineage (Figure 3; Supplementary Table 4). Group B and Group C include clades with MerA sequences from confirmed Hg(II) reducers (Boyd and Barkay, 2012; Supplementary Table 1). These clades have been denoted with yellow stars (Figure 1).

The earliest branching lineage of MerA (Group A) comprises only archaeal sequences, suggesting an origin for MerA among Archaea. Specifically, the archaeal phyla that comprise Group A include MerA from Crenarchaeota (class Thermoprotei; lineage K03), Thaumarchaeota (order Aigarchaeota; K02), Geothermarchaeota (lineage K04), Thaumarchaeota (K05), Euryarchaeota (class Thermoplasmata; K06 and K09), and *Candidatus* Geoarchaeota (K07 and K10). Characterized members of these phyla are thermophiles and often acidophiles and have been identified in a variety of thermal settings including hydrothermal fluids, geothermal springs, and other volcanic-influenced environments (Golyshina, 2011; Kozubal et al., 2013; Reysenbach, 2015; Zillig and Reysenbach, 2015; Jungbluth et al., 2017; Hua et al., 2018). Of the above lineages, MerA activity has been documented in the crenarchaeota *Sulfolobus solfataricus* (Schelert et al., 2004) and *Metallosphaera sedula* (Artz et al., 2015). Apart from the lineages Crenarchaeota and Euryarchaeota that include homologs from aerobic archaea (Golyshina et al., 2009; Kozubal et al., 2011), all thermophilic and hyperthermophilic archaea with MerA that cluster in Group A are putative anaerobes. The most basal lineage comprises both aerobic or facultatively anaerobic crenarchaeota, including MerA from the genera *Pyrobaculum*, *Thermoproteus*, *Thermofilum*, and *Aeropyrum*. This suggests a thermophilic origin for MerA in a volcanically influenced habitat, potentially with low O_2_ availability, a finding that is consistent with previous metagenomic surveys (Geesey et al., 2016), phylogenetic studies of MerA (Barkay et al., 2010; Boyd and Barkay, 2012), and the unusually high optimal temperature for MerA enzymes in a variety of mesophilic bacterial lineages (Vetriani et al., 2005; Barkay et al., *unpublished*). Volcanic habitats are often enriched in naturally high levels of Hg(II) (Varekamp and Buseck, 1986), possibly due to oxidative leaching of Hg-containing minerals (e.g., HgS) in the subsurface. Naturally high levels of Hg(II) in geothermal environments would be expected to be a potent selective pressure to evolve a dedicated Hg(II) detoxification system in the form of MerA. Thus, the present phylogeny, which consists of a much larger data base of MerA sequences (Table 1) than in previously analyses (Barkay et al., 2010; Boyd and Barkay, 2012), has resulted in a refinement of the MerA evolutionary path to firmly establish an origin for MerA among thermophilic Archaea from geothermal environments.

MerA homologs that comprise Group B consist of both archaeal and bacterial homologs that are further separated into 3 subgroups denoted by light blue and purple coloring (Figure 3). The first subgroup consists of MerA homologs from thermophilic and hyperthermophilic euryarchaeal classes Thermococci and Archaeoglobi (K11). This MerA lineage is sister to a lineage that comprises MerA from members of *Candidatus* Daviesbacteria (K12), *Candidatus* Peregrinibacteria (K13), *Candidatus* Giovannonibacteria (K14), and a variety of bacterial MerA homologs that belong to members of the Candidate Phyla Radiation (CPR) recovered from an aquifer close to the Colorado River, United States (Brown et al., 2015; Anantharaman et al., 2016). This suggests genomic acquisition of MerA among a bacterial member of the CPR through lateral gene transfer (LGT) that likely involved a thermophilic archaeon. The second subgroup of MerA homologs in Group B includes lineages from clades K15 to K21 (Figure 3; Supplementary Table 4). The second subgroup of MerA homologs in Group B includes all lineages from clades K15 to K21 (Figure 3; Supplementary Table 4). Clade K15 consists of MerA homologs from euryarchaeal methanogenic and methane-oxidizing Methanomicrobia (Kendall and Boone, 2006; Arshad et al., 2015), while the Thaumarchaeota clade K16 includes homologs from aerobic ammonia-oxidizing *Nitrososphaera* (Kerou and Schleper, 2016). The remaining MerA homologs in this subgroup (K17-K21) are from thermophilic Thermoprotei and Thermoplasmata. The third subgroup in Group B (depicted by orange) is nested within the second archaeal subgroup and includes many bacterial MerA such as the putatively anaerobic gammaproteobacterial group K27 (Figure 3; Magot et al., 1992; Vandieken et al., 2006; Shelobolina et al., 2007; Slobodkina et al., 2012), the anaerobic Kryptonia, isolated from neutral pH thermal springs (Eloe-Fadrosh et al., 2016), the anaerobic Chloroflexi from the *Dehalococcoides* genus (K28; Löffler et al., 2013), and the anaerobic Atribacteria (Liu et al., 2019). Acquisition of MerA among Bacteria comprising the third subgroup of Group B likely occurred through a LGT event involving an archaeon, possibly an ancestor of *Candidatus* Odinarchaeota, *Candidatus* Bathyarcheota, or *Candidatus* Heimdallarchaeota. In summary, multiple cross-domain LGT events are invoked to explain the genomic distribution of MerA that comprise Group B, events that substantially increased the taxonomic and ecological distribution of MerA in natural systems.

Group C of the MerA phylogeny comprises homologs that are primarily bacterial, with the exception of one euryarchaeal lineage (K34; Figure 3). The first subgroup in Group C (colored red; Figure 3) includes MerA from the thermophilic bacterium *Deferribacter desulfuricans* (Takaki et al., 2010) and several MerA homologs from unclassified bacterial lineages (lineage K32) that are reported as microaerophilic thermophiles according to the IMG database. This lineage also includes several MerA sequences from MAGs that are derived from artificial or human-impacted environments (Supplementary Table 2). These include the manganese-oxidizing Nitrospiraceae bacterium Mn-1 isolated from tap water (Yu and Leadbetter, 2020), *Tissierella creatinophila* KRE4 isolated from sewage sludge (Harms et al., 1998), several unclassified deltaproteobacteria (lineage K31) isolated from acid mine drainage (Tan et al., 2019), and several unclassified deltaproteobacteria and unclassified Nitrospirae bacteria (lineages K29 and K30, respectively) from a metal-contaminated aquifer near the Colorado River, Colorado, United States (Hug et al., 2015). This subgroup of Group C also includes MerA from the thermophilic hydrogen- or sulfur-oxidizing lineages of Aquificae (Gupta, 2014), a bacterial lineage where MerA was previously suggested to have originated (Barkay et al., 2010; Boyd and Barkay, 2012). However, Aquificae MerA are nested among several bacterial lineages including unclassified deltaproteobacterial (K29 and K31) and an unclassified *Nitrospira* MerA lineage (K30), all of which derive from MAGs. The discrepancy between the findings of previous studies that showed Aquificae MerA to branch at the base of MerA phylogenies (Barkay et al., 2010; Boyd and Barkay, 2012) and the findings reported herein is almost certainly due to the nearly ten-fold increase in the diversity of archaeal MerA considered in the present study, including those from MAGs and SCGs from environmental samples.

A second subgroup within Group C (colored purple, Figure 3) include MerA from two *Archaeoglobus* (lineage K34; Euryarchaeota) genomes from deep basaltic subseafloor fluids collected from the Juan de Fuca ridge (Jungbluth et al., 2017). Within this subgroup, lineage K34 branches basal to MerA from several Chloroflexi lineages (lineages K35, K36, K39), a Firmicutes bacterium (*Limnochorda pilosa* HC45), Nitrospirae lineage (K37), a lineage comprising unclassified Bacteria (K38), and the Acidobacteria (lineage K40). Apart from several MerA-encoding Chloroflexi genomes that derive from anaerobic aquatic sediments (Löffler et al., 2013; Hug et al., 2015), the rest of the MerA-encoding Chloroflexi lineages are putative thermophiles from geothermal environments (Hedlund et al., 2015; Watanabe et al., 2016; Sakoula et al., 2018; Zheng et al., 2019). The position of Euryarchaeota MerA in the Group C lineage suggests at least two separate cross-domain LGT events possibly took place to genomically acquire MerA as well as to diversify it among members of Group C, with both likely taking place in geothermal environments. The first LGT event likely involved genomic acquisition of MerA in a thermophilic ancestor of Euryarchaeota involving what was likely a thermophilic bacterium from the first subgroup (purple) of Group C, such as an ancestor of the Deferribacteres. The second LGT likely involved transfer of MerA from a thermophilic ancestor of Euryarchaeota to a thermophilic ancestor of Chloroflexi.

The third subgroup of Group C MerA in the phylogeny (red, Figure 3) comprises two predominant lineages of bacteria, both of which include early evolving members of the Proteobacteria (within this subgroup of Group C). Thus, it is possible that MerA in this subgroup was genomically acquired via LGT involving an ancestor of Proteobacteria. Interspersed among proteobacterial MerA are MerA from a variety of bacterial phyla including lineages comprising non-thermophilic members of the Proteobacteria, Actinobacteria, Bacteroidetes, Verrucomicrobia, Chloroflexi, *Candidatus* Rokubacteria, among others. In addition, this subgroup of Group C includes MerA from both thermophilic and non-thermophilic members of the Deinococcus-Thermus lineage. This suggests numerous LGT events have taken place among ancestors of these phyla that have broadened both the taxonomic diversity and ecological distribution of MerA. This is consistent with the existence of selective pressure to acquire and maintain MerA in taxa that inhabit a variety of environment types and supports previous studies that have shown that *mer* genes have a high propensity to be laterally transferred (Bogdanova et al., 1998; Barkay et al., 2003; Ojo et al., 2004), including several instances of cross-domain transfers (Barkay et al., 2010; Boyd and Barkay, 2012).

### Mapping the Taxonomic Distribution of MerB Onto the MerA Phylogeny

Although proteins with TRASH domains (a metal-binding domain involved in heavy metal sensing, trafficking, and resistance) and the NosL protein (accessory protein of the nitrous oxide reductase system) have been suggested to share ancestry with MerB due to structural homology (Ettema et al., 2003; Taubner et al., 2006; Kaur and Subramanian, 2014), alignments revealed very little overlap in or homology among aligned positions (data not shown). As such, these paralogs cannot confidently be used as an outgroup in phylogenetic reconstructions of MerB (Supplementary Figure 2). Other suitable paralogs do not exist in current databases, preventing a directional assessment of the evolution of this protein. As an alternative, the distribution of MerB among MerA-encoding genomes was mapped on the MerA phylogeny (Figure 3). Assuming that MerA is the backbone of the *mer* detoxification system and that other functionalities were recruited later (Boyd and Barkay, 2012), this approach can provide insight into when MerB was recruited to MerA-encoding genomes as a dedicated strategy to detoxify organomercurials and increase the spectrum of mercurial resistance. Importantly, concentrations of MeHg and other organomercury compounds are often below the toxicity limit of cells in most environments. For example, MeHg concentrations even in contaminated environments are at ppt to ppb range (Gilmour et al., 1998) while MeHg toxicity to microbial growth is at the ppb to ppm range (Baldi et al., 1993). This raises the question of whether such compounds served as agents of natural selection as it related to acquisition of MerB and, if not, what the selective pressure to acquire MerB was.

MerB proteins were absent among members of the early branching lineages of crenarchaeal and thaumarchaeal MerA (Group A, Figure 3). Additionally, MerB homologs were not identified among members of the *Candidatus* Geoarchaeota, an archaeal phylum that features multiple genomes that encode MerA homologs (Figure 3; Table 1). The earliest branching MerA homolog in a genome that also encoded MerB was identified in Group B and specifically in a subgroup that includes lineages K15 and K16 (Figure 3; Supplementary Table 4). Lineage K15 includes methanogenic and mesophilic methane-oxidizing Euryarchaeota (Arshad et al., 2015), and lineage K16 includes mesophilic ammonia-oxidizing Thaumarchaeota (Kerou and Schleper, 2016). This suggests that MerB was likely recruited to the *mer* operon in an ancestor of these phyla. Previous findings revealed an increase in *mer*-encoded functionalities over evolutionary time (Boyd and Barkay, 2012). Similarly, early evolving lineages such as Crenarchaeota, Thaumarchaeota, and Geothermarchaeota tend to feature only MerA, while more recently evolved lineages (e.g., Proteobacteria) tend to harbor both MerA and MerB and other ancillary functions to improve Hg tolerance. This may suggest that recruitment of MerB in MerA-encoding organisms occurred after they had diversified into more moderate temperature environments.

Other Group B MerA-encoding lineages that also encode MerB include a member of the archaeal phylum Heimdallarchaeota, a phylum of organisms that have been detected in anaerobic marine sediments (Zaremba-Niedzwiedzka et al., 2017). Additionally in Group C, MerA-encoding genomes also often encode MerB, including a MerA-encoding Nitrospiraceae bacterium isolated from municipal tap water and a MerA-encoding Firmicutes bacterium isolated from sewage sludge (Harms et al., 1998). Many of the natural and artificial environments where organisms encoding both MerA and MerB have been isolated from tend to also harbor microorganisms that encode *hgcAB* genes (Podar et al., 2015; McDaniel et al., 2020). The production of MeHg in these habitats by HgcAB may have been the selective pressure that led to the recruitment of MerB to these organisms allowing for MeHg detoxification. The low (nM) concentrations of MeHg and other organomercurials in most natural habitats (Gilmour et al., 1998; Schartup et al., 2015) may reflect a steady state concentration attributable to near balanced rates of production and degradation.

Early evolving subgroups of Group C MerA include thermophilic Chloroflexi lineages; specifically, members of the Ktedonobacteria class of Chloroflexi (lineage K36) from geothermal hot springs (Tomazini et al., 2019) and Kallotenuaceae members (lineage K39) from the Great Boiling Spring in Nevada (Hedlund et al., 2015). However, these MerA-encoding genomes tend to not encode MerB. High temperature environments like hydrothermal vents and geothermal springs apparently do not favor microbial Hg(II) methylation, based on the limited distribution of *hgcAB* in these environment types (Driscoll et al., 2013; Podar et al., 2015). Despite reports of *hgcAB*, and thus Hg(II) methylation potential, in metagenomes recovered from geothermal environments, none of the organisms that encode these HgcAB can be confidently assigned to thermophiles. For example, while *hgcAB* was identified in marine mats in the submarine Kolumbo volcano (Podar et al., 2015), these mats were collected from a low-temperature zone in this system (Oulas et al., 2015). Furthermore, fused *hgcAB* sequences were identified in the thermophilic archaeon *Pyrococcus furiosus* (Podar et al., 2015). However, this strain was shown to be incapable of methylating Hg(II). Additionally, BLASTp searches of *hgcA* identified a homolog of the gene in a member of the archaeal Thermoplasmatales. However, this microorganism is an anaerobic mesophile isolated from an aquatic environment (Baker et al., 2015). Finally, *hgcA* was identified in the Coriobacteriia class of Actinobacteria and was linked to a geothermal environment because the Coriobacteriia belongs to the OPB41 class (McDaniel et al., 2020). The OPB41 class was first characterized through 16S rRNA gene sequencing of DNA from a hot spring (Hugenholtz et al., 1998). However, members of OPB41 have been identified in numerous non-thermophilic environments, including in cold basaltic sea sediments (Bird et al., 2019). As such, it is unclear if this *hgcAB*-encoding organism is a thermophile.

Collectively, these observations suggest that the aforementioned lineages that encode both MerA and MerB likely recruited MerB in a lower temperature habitat where mercury methylation is more likely to occur (Celo et al., 2006; Martins et al., 2006). Organisms with MerB then radiated among numerous bacterial and archaeal phyla that inhabit natural and anthropogenically impacted environments where Hg(II) is available for anaerobic methylation. On the basis of the distribution and abundance of *hgcAB* genes, such environments predominantly include subsurface aquifer systems, thawing permafrost layers, aquatic sediments, wetlands, and saturated soils (Podar et al., 2015; McDaniel et al., 2020).

## Conclusion

The present study, which included analyses of the distribution and diversity of MerAB in isolate genomes, SCGs, and MAGs, significantly expanded the taxonomic and phylogenetic diversity of homologs when compared to previous studies that analyzed only the genomes of isolates (Barkay et al., 2010; Boyd and Barkay, 2012). This expansion included identifying MerA and/or MerB in genomes that correspond to 55 bacterial and archaeal lineages at the phylum level, including numerous phyla that were not previously reported to encode these functionalities. The expansion of the diversity of taxa that carry the *mer* system clearly points to the need to characterize the Hg transformation activities of a larger and more diverse range of organisms. The insights obtained will improve our understanding of the physiology and ecology of microorganisms that largely modulate the forms and fate of Hg in the environment, their evolution, and their potential uses in Hg bioremediation.

Numerous microbial genomes were identified that encode MerB but not MerA, suggesting the presence of potentially alternative mechanisms to detoxify cytoplasmic Hg(II) formed from demethylation of MeHg. A number of potential mechanisms that allow for Hg(II) detoxification are presented. These are largely based on alternative mechanisms for reduction of Hg(II) and on various aspects of the interactions of Hg with sulfur. Furthermore, the existence of these alternative mechanisms of detoxifying Hg(II) suggest that MerA originated as a superior Hg(II)-reducing catalyst that has been maintained in a diverse array of microbial taxa. The numerous successful LGT events required to explain the widespread taxonomic distribution of MerA across Archaea and Bacteria support this hypothesis. The broad distribution and high occurrence of MerB in the absence of MerA highlights how little we know about this enzyme and its role as a catalyst in the *mer* system. Moreover, in light of the scarcity of MerB paralogs, untangling the path of its evolution is challenging. Further, it is not clear what selective pressure might have shaped the origin and evolution of MerB considering the low concentrations of MeHg in the natural environment (although, that concentration might be higher immediately proximal to methylating microbes – *see* below). Thus, further exploration of MerB homologs, including those with sequence variations of unknown functional consequences, in diverse taxa from a variety of environmental settings (e.g., low versus high temperature) is warranted.

With an expanded database of MerA homologs and a robust phylogenetic reconstruction of these homologs, we present a refined path for the evolution of MerA that improves our understanding of the origin, evolution, and environmental distribution of this enzyme. Firstly, the earliest evolving MerA proteins were identified among the genomes of organisms inhabiting high temperature hydrothermal environments, which is consistent with previous studies (Barkay et al., 2010; Boyd and Barkay, 2012). It is possible that naturally elevated concentrations of Hg(II) that typify hydrothermal environments acted as the original selective pressure to evolve this enzyme. However, the earliest branching MerA sequences were from thermophilic Archaea rather than Bacteria, which differs from the origin for MerA among bacterial and thermophilic Aquificales as advocated previously (Barkay et al., 2010; Boyd and Barkay, 2012). The cultivation-independent nature of the SCGs and MAGs included in the study reported here, which resulted in a 25-fold increase in the number of MerA homologs known, is almost certainly responsible for the change in the topology of the tree. Nonetheless, like previous phylogenetic studies of MerA, numerous instances of cross domain and cross phylum LGT events were observed in the phylogenetic reconstruction conducted herein. Combined with the finding of multiple (up to 8 copies) of MerA in the genomes of some organisms and the detection of MerA on plasmids (which are likely to be underreported for reasons outlined above), these data point to the fitness advantage that MerA-encoding cells have in Hg(II) contaminated environments and the selective pressure for cells to maintain MerA once it is acquired via LGT. The duplication of MerA encoding genes may have allowed for cells to increase their responsiveness to Hg(II) toxicity (gene dosage effect) or to respond to Hg(II) toxicity with an enzyme with slightly altered kinetics or substrate specificity (sub-functionalization).

While it was not possible to perform an analysis of the evolution of MerB using a rooted phylogeny, mapping of MerB on the MerA phylogeny suggests that it was likely recruited to the *mer* operon relatively recently. Furthermore, the near universal absence of MerB homologs among thermophiles, when combined with the fact that the earliest evolving MerA-encoding genome that also encodes MerB derives from a mesophile, suggests that the ability to demethylate MeHg emerged in a lower temperature setting following the emergence of *mer* systems in mesophiles. This is potentially consistent with the limited distribution of genes involved in biotic MeHg production (i.e., *hgcAB*) in thermophiles or high temperature habitats but being pervasive in lower temperature habitats (Podar et al., 2015; McDaniel et al., 2020). Consistent with this notion, MerB-encoding genomes were identified among organisms or DNA recovered from a variety of habitat types that likely favor MeHg production, including aquatic sediments, thawing permafrosts, subsurface aquifers, and soils. This may suggest a relationship between the biological production and consumption of MeHg in natural habitats. The expanded taxonomic diversity of MerB homologs and the metabolic backgrounds of the organisms that they derive from may provide new strategies that could be employed in the management of MeHg contamination and its bioremediation.

## Data Availability Statement

The raw data supporting the conclusions of this article will be made available by the authors, without undue reservation.

## Author Contributions

CAC and ESB designed the study. CAC carried out bioinformatic and phylogenetic analyses. All authors contributed to data analysis and the writing of the manuscript.

## Conflict of Interest

The authors declare that the research was conducted in the absence of any commercial or financial relationships that could be construed as a potential conflict of interest.

## References

[B1] Anantharaman K. Breier J. A. Dick G. J. (2016). Metagenomic resolution of microbial functions in deep-sea hydrothermal plumes across the Eastern Lau Spreading Center. *ISME J.* 10 225–239. 10.1038/ismej.2015.81 26046257 PMC4681857

[B2] Antipov D. Raiko M. Lapidus A. Pevzner P. A. (2019). Plasmid detection and assembly in genomic and metagenomic data sets. *Genome Res.* 29 961–968. 10.1101/gr.241299.118 31048319 PMC6581055

[B3] Ariza M. E. Williams M. V. (1999). Lead and mercury mutagenesis: type of mutation dependent upon metal concentration. *J. Biochem. Mol. Toxicol.* 13 107–112. 9890195 10.1002/(sici)1099-0461(1999)13:2<107::aid-jbt6>3.0.co;2-0

[B4] Arshad A. Speth D. R. De Graaf R. M. Op den Camp H. J. M. Jetten M. S. M. Welte C. U. (2015). A metagenomics-based metabolic model of nitrate-dependent anaerobic oxidation of methane by Methanoperedens-like *Archaea*. *Front. Microbiol.* 6:1423. 10.3389/fmicb.2015.01423 26733968 PMC4683180

[B5] Artz J. H. White S. N. Zadvornyy O. A. Fugate C. J. Hicks D. Gauss G. H. (2015). Biochemical and structural properties of a thermostable mercuric ion reductase from *Metallosphaera sedula*. *Front. Bioeng. Biotechnol.* 3:97. 10.3389/fbioe.2015.00097 26217660 PMC4500099

[B6] Baker B. J. Lazar C. S. Teske A. P. Dick G. J. (2015). Genomic resolution of linkages in carbon, nitrogen, and sulfur cycling among widespread estuary sediment bacteria. *Microbiome* 3:14. 10.1186/s40168-015-0077-6 25922666 PMC4411801

[B7] Baldi F. Pepi M. Filippelli M. (1993). Methylmercury resistance in *Desulfovibrio desulfuricans* strains in relation to methylmercury degradation. *Appl. Environ. Microbiol.* 59 2479–2485. 10.1128/aem.59.8.2479-2485.1993 16349013 PMC182309

[B8] Barkay T. Kritee K. Boyd E. Geesey G. (2010). A thermophilic bacterial origin and subsequent constraints by redox, light and salinity on the evolution of the microbial mercuric reductase. *Environ. Microbiol.* 12 2904–2917. 10.1111/j.1462-2920.2010.02260.x 20545753

[B9] Barkay T. Miller S. M. Summers A. O. (2003). Bacterial mercury resistance from atoms to ecosystems. *FEMS Microbiol. Rev.* 27 355–384. 10.1016/S0168-6445(03)00046-9 12829275

[B10] Ben-Bassat D. Mayer A. M. (1978). Light-induced Hg volatilization and O2 evolution in *Chlorella* and the effect of DCMU and methylamine. *Physiol. Plant.* 42 33–38. 10.1111/j.1399-3054.1978.tb01534.x

[B11] Benedikter B. J. Weseler A. R. Wouters E. F. M. Savelkoul P. H. M. Rohde G. G. U. Stassen F. R. M. (2018). Redox-dependent thiol modifications: implications for the release of extracellular vesicles. *Cell. Mol. Life Sci.* 75 2321–2337. 10.1007/s00018-018-2806-z 29594387 PMC5986851

[B12] Benison G. C. Di Lello P. Shokes J. E. Cosper N. J. Scott R. A. Legault P. (2004). A stable mercury-containing complex of the organomercurial lyase MerB: catalysis, product release, and direct transfer to MerA. *Biochemistry* 43 8333–8345. 10.1021/bi049662h 15222746

[B13] Benoit J. M. Gilmour C. C. Mason R. P. Riedel G. S. Riedel G. F. (1998). Behavior of mercury in the Patuxent river estuary. *Biogeochemistry* 40 249–265. 10.1023/a:1005905700864

[B14] Bird J. T. Tague E. D. Zinke L. Schmidt J. M. Steen A. D. Reese B. (2019). Uncultured microbial phyla suggest mechanisms for multi-thousand-year subsistence in Baltic Sea sediments. *mBio* 10:e2376-18. 10.1128/mbio.02376-18 30992358 PMC6469976

[B15] Bogdanova E. S. Bass I. A. Minakhin L. S. Petrova M. A. Mindlin S. Z. Volodin A. A. (1998). Horizontal spread of mer operons among Gram-positive bacteria in natural environments. *Microbiology* 144 609–620. 10.1099/00221287-144-3-609 9534232

[B16] Böhm M.-E. Huptas C. Krey V. M. Scherer S. (2015). Massive horizontal gene transfer, strictly vertical inheritance and ancient duplications differentially shape the evolution of *Bacillus cereus* enterotoxin operons hbl, cytK and nhe. *BMC Evol. Biol.* 15:246. 10.1186/s12862-015-0529-4 26555390 PMC4641410

[B17] Bowers R. M. Kyrpides N. C. Stepanauskas R. Harmon-Smith M. Doud D. Reddy T. B. K. (2017). Minimum information about a single amplified genome (MISAG) and a metagenome-assembled genome (MIMAG) of Bacteria and *Archaea*. *Nat. Biotechnol.* 35 725–731. 10.1038/nbt.3893 28787424 PMC6436528

[B18] Boyd E. S. Amenabar M. J. Poudel S. Templeton A. S. (2020). Bioenergetic constraints on the origin of autotrophic metabolism. *Philos. Trans. R. Soc. A Math. Phys. Eng. Sci.* 378:20190151. 10.1098/rsta.2019.0151 31902344 PMC7015307

[B19] Boyd E. S. Barkay T. (2012). The mercury resistance operon: from an origin in a geothermal environment to an efficient detoxification machine. *Front. Microbiol.* 3:349. 10.3389/fmicb.2012.00349 23087676 PMC3466566

[B20] Boyd E. S. King S. Tomberlin J. K. Nordstrom D. K. Krabbenhoft D. P. Barkay T. (2009). Methylmercury enters an aquatic food web through acidophilic microbial mats in Yellowstone National Park, Wyoming. *Environ. Microbiol.* 11 950–959. 10.1111/j.1462-2920.2008.01820.x 19170726

[B21] Brown C. T. Hug L. A. Thomas B. C. Sharon I. Castelle C. J. Singh A. (2015). Unusual biology across a group comprising more than 15% of domain Bacteria. *Nature* 523 208–211. 10.1038/nature14486 26083755

[B22] Buckel W. Thauer R. K. (2018). Flavin-based electron bifurcation, ferredoxin, flavodoxin, and anaerobic respiration with protons (Ech) or NAD+ (Rnf) as electron acceptors: a historical review. *Front. Microbiol.* 9:401. 10.3389/fmicb.2018.00401 29593673 PMC5861303

[B23] Celo V. Lean D. R. S. Scott S. L. (2006). Abiotic methylation of mercury in the aquatic environment. *Sci. Total Environ.* 368 126–137. 10.1016/j.scitotenv.2005.09.043 16226793

[B24] Chen I. M. A. Chu K. Palaniappan K. Pillay M. Ratner A. Huang J. (2018). IMG/M v.5.0: an integrated data management and comparative analysis system for microbial genomes and microbiomes. *Nucleic Acids Res.* 47 D666–D677. 10.1093/nar/gky901 30289528 PMC6323987

[B25] Chen L. X. Méndez-García C. Dombrowski N. Servín-Garcidueñas L. E. Eloe-Fadrosh E. A. Fang B. Z. (2018). Metabolic versatility of small *Archaea* Micrarchaeota and Parvarchaeota. *ISME J.* 12 756–775. 10.1038/s41396-017-0002-z 29222443 PMC5864196

[B26] Chien M.-F. Narita M. Lin K.-H. Matsui K. Huang C.-C. Endo G. (2010). Organomercurials removal by heterogeneous merB genes harboring bacterial strains. *J. Biosci. Bioeng.* 110 94–98. 10.1016/j.jbiosc.2010.01.010 20541123

[B27] Christensen G. A. Gionfriddo C. M. King A. J. Moberly J. G. Miller C. L. Somenahally A. C. (2019). Determining the reliability of measuring mercury cycling gene abundance with correlations with mercury and methylmercury concentrations. *Environ. Sci. Technol.* 53 8649–8663. 10.1021/acs.est.8b06389 31260289

[B28] Clarkson T. W. Magos L. (2006). The toxicology of mercury and its chemical compounds. *Crit. Rev. Toxicol.* 36 609–662. 10.1080/10408440600845619 16973445

[B29] Colman D. R. Poudel S. Hamilton T. L. Havig J. R. Selensky M. J. Shock E. L. (2018). Geobiological feedbacks and the evolution of thermoacidophiles. *ISME J.* 12 225–236. 10.1038/ismej.2017.162 29028004 PMC5739016

[B30] Compeau G. C. Bartha R. (1985). Sulfate-reducing bacteria: principal methylators of mercury in anoxic estuarine sediment. *Microbiology* 50 498–502. 10.1128/aem.50.2.498-502.1985 PMC238649 16346866

[B31] Costa M. Liss P. S. (1999). Photoreduction of mercury in sea water and its possible implications for Hg0 air-sea fluxes. *Mar. Chem.* 68 87–95. 10.1016/S0304-4203(99)00067-5

[B32] Di Lello P. Benison G. C. Valafar H. Pitts K. E. Summers A. O. Legault P. (2004). NMR structural studies reveal a novel protein fold for MerB, the organomercurial lyase involved in the bacterial mercury resistance system. *Biochemistry* 43 8322–8332. 10.1021/bi049669z 15222745

[B33] Diels L. Mergeay M. (1990). DNA probe-mediated detection of resistant Bacteria from soils highly polluted by heavy metals. *Appl. Environ. Microbiol.* 56 1485–1491. 10.1128/AEM.56.5.1485-1491.1990 16348196 PMC184435

[B34] Driscoll C. T. Mason R. P. Chan H. M. Jacob D. J. Pirrone N. (2013). Mercury as a global pollutant: sources, pathways, and effects. *Environ. Sci. Technol.* 47 4967–4983. 10.1021/es305071v 23590191 PMC3701261

[B35] Eloe-Fadrosh E. A. Ivanova N. N. Woyke T. Kyrpides N. C. (2016). Metagenomics uncovers gaps in amplicon-based detection of microbial diversity. *Nat. Microbiol.* 1:15032. 10.1038/nmicrobiol.2015.32 27572438

[B36] Essa A. M. M. Macaskie L. E. Brown N. L. (2002). Mechanisms of mercury bioremediation. *Biochem. Soc. Trans.* 30 672–674. 10.1042/BST0300672 12196161

[B37] Essa A. M. M. Macaskie L. E. Brown N. L. (2005). A new method for mercury removal. *Biotechnol. Lett.* 27 1649–1655. 10.1007/s10529-005-2722-9 16247669

[B38] Ettema T. J. G. Huynen M. A. de Vos W. M. van der Oost J. (2003). TRASH: a novel metal-binding domain predicted to be involved in heavy-metal sensing, trafficking, and resistance. *Trends Biochem. Sci.* 28 170–173. 10.1016/S0968-0004(03)00037-9 12713899

[B39] Fallon E. K. Petersen S. Brooker R. A. Scott T. B. (2017). Oxidative dissolution of hydrothermal mixed-sulphide ore: an assessment of current knowledge in relation to seafloor massive sulphide mining. *Ore Geol. Rev.* 86 309–337. 10.1016/j.oregeorev.2017.02.028

[B40] Fleming E. J. Mack E. E. Green P. G. Nelson D. C. (2006). Mercury methylation from unexpected sources: molybdate-inhibited freshwater sediments and an iron-reducing bacterium. *Appl. Environ. Microbiol.* 72 457–464. 10.1128/AEM.72.1.457-464.2006 16391078 PMC1352261

[B41] Geesey G. G. Barkay T. King S. (2016). Microbes in mercury-enriched geothermal springs in western North America. *Sci. Total Environ.* 569–570 321–331. 10.1016/j.scitotenv.2016.06.080 27344121

[B42] Gilmour C. C. Bullock A. L. McBurney A. Podar M. Elias D. A. (2018). Robust mercury methylation across diverse methanogenic *Archaea*. *mBio* 9:e2403-17. 10.1128/mBio.02403-17 29636434 PMC5893877

[B43] Gilmour C. C. Henry E. A. (1991). Mercury methylation in aquatic systems affected by acid deposition. *Environ. Pollut.* 71 131–169. 10.1016/0269-7491(91)90031-Q 15092118

[B44] Gilmour C. C. Henry E. A. Ralph M. (1992). Sulfate stimulation of mercury methylation in freshwater sediments. *Environ. Sci. Technol.* 26 2281–2287. 10.1021/es00035a029

[B45] Gilmour C. C. Podar M. Bullock A. L. Graham A. M. Brown S. D. Somenahally A. C. (2013). Mercury methylation by novel microorganisms from new environments. *Environ. Sci. Technol.* 47 11810–11820. 10.1021/es403075t 24024607

[B46] Gilmour C. C. Riedel G. S. Ederington M. C. Bell J. T. Benoit J. M. (1998). Methylmercury concentrations and production rates across a trophic gradient in the northern Everglades. *Biogeochemistry* 40 327–345. 10.1023/A:1005972708616

[B47] Gionfriddo C. M. Tate M. T. Wick R. R. Schultz M. B. Zemla A. Thelen M. P. (2016). Microbial mercury methylation in Antarctic sea ice. *Nat. Microbiol.* 1:16127. 10.1038/nmicrobiol.2016.127 27670112

[B48] Glendinning K. J. Macaskie L. E. Brown N. L. (2005). Mercury tolerance of thermophilic Bacillus sp. and Ureibacillus sp. *Biotechnol. Lett.* 27 1657–1662. 10.1007/s10529-005-2723-8 16247670

[B49] Golyshina O. V. (2011). Environmental, biogeographic, and biochemical patterns of *Archaea* of the family *Ferroplasmaceae*. *Appl. Environ. Microbiol.* 77 5071–5078. 10.1128/AEM.00726-11 21685165 PMC3147448

[B50] Golyshina O. V. Lünsdorf H. Kublanov I. V. Goldenstein N. I. Hinrichs K. U. Golyshin P. N. (2016). The novel extremely acidophilic, cell-wall-deficient archaeon *Cuniculiplasma divulgatum* gen. Nov., sp. nov. represents a new family, *Cuniculiplasmataceae* fam. nov., of the order *Thermoplasmatales*. *Int. J. Syst. Evol. Microbiol.* 66 332–340. 10.1099/ijsem.0.000725 26518885 PMC4806541

[B51] Golyshina O. V. Yakimov M. M. Lunsdorf H. Ferrer M. Nimtz M. Timmis K. N. (2009). *Acidiplasma aeolicum* gen. nov., sp. nov., a euryarchaeon of the family *Ferroplasmaceae* isolated from a hydrothermal pool, and transfer of *Ferroplasma cupricumulans* to *Acidiplasma cupricumulans* comb. nov. *Int. J. Syst. Evol. Microbiol.* 59 2815–2823. 10.1099/ijs.0.009639-0 19628615

[B52] Goris J. De Vos P. Coenye T. Hoste B. Janssens D. Brim H. (2001). Classification of metal-resistant bacteria from industrial biotopes as *Ralstonia campinensis* sp. nov., *Ralstonia metallidurans* sp. nov. and *Ralstonia basilensis* Steinle et al. 1998 emend. *Int. J. Syst. Evol. Microbiol.* 51 1773–1782. 10.1099/00207713-51-5-1773 11594608

[B53] Grégoire D. S. Lavoie N. C. Poulain A. J. (2018). Heliobacteria reveal fermentation as a key pathway for mercury reduction in anoxic environments. *Environ. Sci. Technol.* 52 4145–4153. 10.1021/acs.est.8b00320 29514452

[B54] Gregoire D. S. Poulain A. J. (2016). A physiological role for Hg II during phototrophic growth. *Nat. Geosci.* 9 121–125. 10.1038/ngeo2629

[B55] Grégoire D. S. Poulain A. J. (2018). Shining light on recent advances in microbial mercury cycling. *Facets* 3 858–879. 10.1139/facets-2018-0015 33356898

[B56] Gu B. Bian Y. Miller C. L. Dong W. Jiang X. Liang L. (2011). Mercury reduction and complexation by natural organic matter in anoxic environments. *Proc. Natl. Acad. Sci. U. S. A.* 108 1479–1483. 10.1073/pnas.1008747108 21220311 PMC3029776

[B57] Gupta A. Phung L. T. Chakravarty L. Silver S. (1999). Mercury resistance in *Bacillus cereus* RC607: transcriptional organization and two new open reading frames. *J. Bacteriol.* 181 7080–7086. 10.1128/JB.181.22.7080-7086.1999 10559175 PMC94184

[B58] Gupta R. S. (2014). “The phylum aquificae,” in *The Prokaryotes – Other Major Lineages of Bacteria and the*, eds Falkow S. (Berlin: Springer-Verlag), 417–445. 10.1007/0-387-30744-3

[B59] Hamelin S. Amyot M. Barkay T. Wang Y. Planas D. (2011). Methanogens: principal methylators of mercury in lake periphyton. *Environ. Sci. Technol.* 45 7693–7700. 10.1021/es2010072 21875053

[B60] Harms C. Schleicher A. Collins M. D. Andreesen J. R. (1998). Tissierella creatinophila sp. nov., a Gram-positive, anaerobic, non-spore-forming, creatinine-fermenting organism. *Int. J. Syst. Bacteriol.* 48 983–993. 10.1099/00207713-48-3-983 9734055

[B61] Hedlund B. P. Murugapiran S. K. Huntemann M. Clum A. Pillay M. Palaniappan K. (2015). High-quality draft genome sequence of *Kallotenue papyrolyticum* JKG1T reveals broad heterotrophic capacity focused on carbohydrate and amino acid metabolism. *Genome Announc.* 3 14–15. 10.1128/genomeA.01410-15 26634758 PMC4669399

[B62] Hoang D. T. Chernomor O. von Haeseler A. Minh B. Q. Vinh L. S. (2018). UFBoot2: improving the ultrafast bootstrap approximation. *Mol. Biol. Evol.* 35 518–522. 10.1093/molbev/msx281 29077904 PMC5850222

[B63] Hu H. Lin H. Zheng W. Rao B. Feng X. Liang L. (2013). Mercury reduction and cell-surface adsorption by *Geobacter sulfurreducens* PCA. *Environ. Sci. Technol.* 47 10922–10930. 10.1021/es400527m 24020841

[B64] Hua Z.-S. Qu Y.-N. Zhu Q. Zhou E.-M. Qi Y.-L. Yin Y.-R. (2018). Genomic inference of the metabolism and evolution of the *Archaeal* phylum Aigarchaeota. *Nat. Commun.* 9:2832. 10.1038/s41467-018-05284-4 30026532 PMC6053391

[B65] Huang C. C. Narita M. Yamagata T. Endo G. (1999). Identification of three merB genes and characterization of a broad- spectrum mercury resistance module encoded by a class II transposon of Bacillus megaterium strain MB1. *Gene* 239 361–366. 10.1016/S0378-1119(99)00388-1 10548738

[B66] Hug L. A. Thomas B. C. Brown C. T. Frischkorn K. R. Williams K. H. Tringe S. G. (2015). Aquifer environment selects for microbial species cohorts in sediment and groundwater. *ISME J.* 9 1846–1856. 10.1038/ismej.2015.2 25647349 PMC4511941

[B67] Hugenholtz P. Pitulle C. Hershberger K. L. Pace N. R. (1998). Novel division level bacterial diversity in a yellowstone hot spring. *J. Bacteriol.* 180 366–376. 10.1128/JB.180.2.366-376.1998 9440526 PMC106892

[B68] Hülter N. Ilhan J. Wein T. Kadibalban A. S. Hammerschmidt K. Dagan T. (2017). An evolutionary perspective on plasmid lifestyle modes. *Curr. Opin. Microbiol.* 38 74–80. 10.1016/j.mib.2017.05.001 28538166

[B69] Jungbluth S. P. Amend J. P. Rappé M. S. (2017). Metagenome sequencing and 98 microbial genomes from juan de fuca ridge flank subsurface fluids. *Sci. Data* 4:170080. 10.1038/sdata.2017.37 28350381 PMC5369317

[B70] Kalyaanamoorthy S. Minh B. Q. Wong T. K. F. von Haeseler A. Jermiin L. S. (2017). ModelFinder: fast model selection for accurate phylogenetic estimates. *Nat. Methods* 14 587–589. 10.1038/nmeth.4285 28481363 PMC5453245

[B71] Kaur G. Subramanian S. (2014). Repurposing TRASH: emergence of the enzyme organomercurial lyase from a non-catalytic zinc finger scaffold. *J. Struct. Biol.* 188 16–21. 10.1016/j.jsb.2014.09.001 25220669

[B72] Kendall M. M. Boone D. R. (2006). “The order methanosarcinales,” in *The Prokaryotes*, eds Dworkin M. Falkow S. Rosenberg E. Schleifer K. H. Stackebrandt E. (New York, NY: Springer), 244–256. 10.1007/0-387-30743-5_12

[B73] Kerou M. Schleper C. (2016). “Nitrososphaera,” in *Bergey’s Manual of Systematics of Archaea and Bacteria*, eds Trujillo M. E. Dedysh S. Kämpfer P. DeVos B. Hedlund P. Rainey F. A. (Hoboken, NJ: Wiley), 1–10. 10.1002/9781118960608.gbm01294

[B74] Kitabatake M. So M. W. Tumbula D. L. Söll D. (2000). Cysteine biosynthesis pathway in the archaeon *Methanosarcina barkeri* encoded by acquired bacterial genes? *J. Bacteriol.* 182 143–145. 10.1128/JB.182.1.143-145.2000 10613873 PMC94250

[B75] Kiyono M. Omura T. Fujimori H. Pan-Hou H. (1995). Organomercurial resistance determinants in *Pseudomonas* K-62 are present on two plasmids. *Arch. Microbiol.* 163 242–247. 10.1007/BF00393375 7763132

[B76] Kondrashov F. A. (2012). Gene duplication as a mechanism of genomic adaptation to a changing environment. *Proc. R. Soc. B Biol. Sci.* 279 5048–5057. 10.1098/rspb.2012.1108 22977152 PMC3497230

[B77] Kozubal M. A. Dlakić M. Macur R. E. Inskeep W. P. (2011). Terminal oxidase diversity and function in “ *Metallosphaera* yellowstonensis ”: gene expression and protein modeling suggest mechanisms of Fe(II) oxidation in the *Sulfolobales*. *Appl. Environ. Microbiol.* 77 1844–1853. 10.1128/AEM.01646-10 21239558 PMC3067268

[B78] Kozubal M. A. Romine M. Jennings R. D. Jay Z. J. Tringe S. G. Rusch D. B. (2013). Geoarchaeota: a new candidate phylum in the *Archaea* from high-temperature acidic iron mats in Yellowstone National Park. *ISME J.* 7 622–634. 10.1038/ismej.2012.132 23151644 PMC3578567

[B79] Kritee K. Motta L. C. Blum J. D. Tsui M. T.-K. Reinfelder J. R. (2018). Photomicrobial visible light-induced magnetic mass independent fractionation of mercury in a marine microalga. *ACS Earth Sp. Chem.* 2 432–440. 10.1021/acsearthspacechem.7b00056

[B80] Lafrance-Vanasse J. Lefebvre M. Lello P. Di Sygusch J. Omichinski J. G. (2009). Crystal structures of the organomercurial lyase MerB in its free and mercury-bound forms: insights into the mechanism of methylmercury degradation. *J. Biol. Chem.* 284 938–944. 10.1074/jbc.M807143200 19004822

[B81] Ledwidge R. Patel B. Dong A. Fiedler D. Falkowski M. Zelikova J. (2005). NmerA, the metal binding domain of mercuric ion reductase, removes Hg2+ from proteins, delivers it to the catalytic core, and protects cells under glutathione-depleted conditions. *Biochemistry* 44 11402–11416. 10.1021/bi050519d 16114877

[B82] Lee S. Kim D. H. Kim K. W. (2018). The enhancement and inhibition of mercury reduction by natural organic matter in the presence of *Shewanella* oneidensis MR-1. *Chemosphere* 194 515–522. 10.1016/j.chemosphere.2017.12.007 29241125

[B83] Lefebvre D. D. Kelly D. Budd K. (2007). Biotransformation of Hg(II) by *Cyanobacteria*. *Appl. Environ. Microbiol.* 73 243–249. 10.1128/AEM.01794-06 17071784 PMC1797140

[B84] Letunic I. Bork P. (2019). Interactive Tree Of Life (iTOL) v4: recent updates and new developments. *Nucleic Acids Res.* 47 W256–W259. 10.1093/nar/gkz239 30931475 PMC6602468

[B85] Lin C.-C. Yee N. Barkay T. (2011). “Microbial transformations in the mercury cycle,” in *Environmental Chemistry and Toxicology of Mercury*, eds G. Liu, Y. Cai, and N. O’Driscoll (Hoboken, NJ: John Wiley & Sons, Inc), 155–191. 10.1002/9781118146644.ch5

[B86] Liu S. Wiatrowski H. A. (2018). Reduction of Hg(II) to Hg(0) by biogenic magnetite from two magnetotactic Bacteria. *Geomicrobiol. J.* 35 198–208. 10.1080/01490451.2017.1362076

[B87] Liu T. Chen D. Li X. Li F. (2019). Microbially mediated coupling of nitrate reduction and Fe(II) oxidation under anoxic conditions. *FEMS Microbiol. Ecol.* 95:fiz030. 10.1093/femsec/fiz030 30844067

[B88] Löffler F. E. Yan J. Ritalahti K. M. Adrian L. Edwards E. A. Konstantinidis K. T. (2013). *Dehalococcoides mccartyi* gen. nov., sp. nov., obligately organohalide-respiring anaerobic bacteria relevant to halogen cycling and bioremediation, belong to a novel bacterial class, *Dehalococcoidia classis* nov., order *Dehalococcoidale*s ord. nov. and famil. *Int. J. Syst. Evol. Microbiol.* 63 625–635. 10.1099/ijs.0.034926-0 22544797

[B89] Luo H. Cheng Q. Pan X. (2020). Photochemical behaviors of mercury (Hg) species in aquatic systems: a systematic review on reaction process, mechanism, and influencing factor. *Sci. Total Environ.* 720:137540. 10.1016/j.scitotenv.2020.137540 32143045

[B90] Maged M. Hosseiny A. El Saadeldin M. K. Aziz R. K. Ramadan E. (2019). Thermal stability of a mercuric reductase from the Red Sea Atlantis II hot brine environment as analyzed by site-directed mutagenesis. *Appl. Environ. Microbiol.* 85:e02387. 10.1128/AEM.02387-18 30446558 PMC6344611

[B91] Magot M. Caumette P. Desperrier J. M. Matheron R. Dauga C. Grimont F. (1992). Desulfovibrio longus sp. nov., a sulfate-reducing bacterium isolated from an oil-producing well. *Int. J. Syst. Bacteriol.* 42 398–402. 10.1099/00207713-42-3-398 1380287

[B92] Maguire F. Jia B. Gray K. L. Lau W. Y. V. Beiko R. G. Brinkman F. S. L. (2020). Metagenome-assembled genome binning methods with short reads disproportionately fail for plasmids and genomic Islands. *Microb. Genomics* 6:mgen000436. 10.1099/mgen.0.000436 33001022 PMC7660262

[B93] Markowitz V. M. Korzeniewski F. Palaniappan K. Szeto E. Werner G. Padki A. (2006). The integrated microbial genomes (IMG) system. *Nucleic Acids Res.* 34 D344–D348. 10.1093/nar/gkj024 16381883 PMC1347387

[B94] Marteyn B. Domain F. Legrain P. Chauvat F. Cassier-Chauvat C. (2009). The thioredoxin reductase-glutaredoxins-ferredoxin crossroad pathway for selenate tolerance in *Synechocystis* PCC6803. *Mol. Microbiol.* 71 520–532. 10.1111/j.1365-2958.2008.06550.x 19040637

[B95] Marteyn B. Sakr S. Farci S. Bedhomme M. Chardonnet S. Decottignies P. (2013). The *Synechocystis* PCC6803 MerA-like enzyme operates in the reduction of both mercury and uranium under the control of the glutaredoxin 1 enzyme. *J. Bacteriol.* 195 4138–4145. 10.1128/JB.00272-13 23852862 PMC3754753

[B96] Martins I. Costa V. Porteiro F. Colaço A. Santos R. (2006). Mercury concentrations in fish species caught at Mid-Atlantic Ridge hydrothermal vent fields. *Mar. Ecol. Prog. Ser.* 320 253–258. 10.3354/meps320253

[B97] Mason R. P. Morel F. M. M. Hemond H. F. (1995). The role of microorganisms in elemental mercury formation in natural waters. *Water Air Soil Pollut.* 80 775–787. 10.1007/BF01189729

[B98] Mason R. P. Reinfelder J. R. Morel F. M. M. (1996). Uptake, toxicity, and trophic transfer of mercury in a coastal diatom. *Environ. Sci. Technol.* 30 1835–1845. 10.1021/es950373d

[B99] Matsui K. Yoshinami S. Narita M. Chien M. F. Phung L. T. Silver S. (2016). Mercury resistance transposons in Bacilli strains from different geographical regions. *FEMS Microbiol. Lett.* 363:fnw013. 10.1093/femsle/fnw013 26802071

[B100] McDaniel E. A. Peterson B. D. Stevens S. L. R. Tran P. Q. Anantharaman K. McMahon K. D. (2020). Expanded phylogenetic diversity and metabolic flexibility of mercury-methylating microorganisms. *mSystems* 5:e299-20. 10.1128/mSystems.00299-20 32817383 PMC7438021

[B101] Miller D. M. Lund B. O. Woods J. S. (1991). Reactivity of Hg(II) with superoxide: evidence for the catalytic dismutation of superoxide by Hg(II). *J. Biochem. Toxicol.* 6 293–298. 10.1002/jbt.2570060409 1663557

[B102] Møller A. K. Barkay T. Hansen M. A. Norman A. Hansen L. H. Sørensen S. J. (2014). Mercuric reductase genes (merA) and mercury resistance plasmids in High Arctic snow, freshwater and sea-ice brine. *FEMS Microbiol. Ecol.* 87 52–63. 10.1111/1574-6941.12189 23909591

[B103] Monecke T. Petersen S. Hannington M. D. Grant H. Samson I. M. (2016). “The minor element endowment of modern sea-floor massive sulfides and comparison with deposits hosted in ancient volcanic successions,” in *Rare Earth and Critical Elements in Ore Deposits*, eds Verplanck P. L. Hitzman M. W. (Littleton, CO: Society of Economic Geologists), 10.5382/Rev.18.11

[B104] Monsieurs P. Provoost A. Mijnendonckx K. Leys N. Gaudreau C. Van Houdt R. (2013). Genome sequence of *Cupriavidus* metallidurans strain H1130, isolated from an invasive human infection. *Genome Announc.* 1 5–6. 10.1128/genomeA.01051-13 24336383 PMC3861436

[B105] Mukai T. Crnković A. Umehara T. Ivanova N. N. Kyrpides N. C. Söll D. (2017). RNA-dependent cysteine biosynthesis in Bacteria and *Archaea*. *mBio* 8:e561-17. 10.1128/mBio.00561-17 28487430 PMC5424206

[B106] Nguyen L.-T. T. Schmidt H. A. von Haeseler A. Minh B. Q. (2015). IQ-TREE: a fast and effective stochastic algorithm for estimating maximum-likelihood phylogenies. *Mol. Biol. Evol.* 32 268–274. 10.1093/molbev/msu300 25371430 PMC4271533

[B107] Norambuena J. Hanson T. E. Barkay T. Boyd J. M. (2019). Superoxide dismutase and pseudocatalase increase tolerance to Hg(II) in *Thermus thermophilus* HB27 by maintaining the reduced bacillithiol pool. *mBio* 10:e183-19. 10.1128/mBio.00183-19 30940703 PMC6445937

[B108] Norambuena J. Wang Y. Hanson T. Boyd J. M. Barkay T. (2018). Low-molecular-weight thiols and thioredoxins are important players in Hg(II) resistance in *Thermus thermophilus* HB27. *Appl. Environ. Microbiol.* 84:e1931-17. 10.1128/AEM.01931-17 29150497 PMC5752852

[B109] Obrist D. Kirk J. L. Zhang L. Sunderland E. M. Jiskra M. Selin N. E. (2018). A review of global environmental mercury processes in response to human and natural perturbations: changes of emissions, climate, and land use. *Ambio* 47 116–140. 10.1007/s13280-017-1004-9 29388126 PMC5794683

[B110] Ojo K. Tung D. Luis H. Bernardo M. Leitao J. Roberts M. (2004). Gram-positive gene in gram-negative oral and urine bacteria. *FEMS Microbiol. Lett.* 238 411–416. 10.1016/j.femsle.2004.08.004 15358427

[B111] Oulas A. Pavloudi C. Polymenakou P. Pavlopoulos G. A. Papanikolaou N. Kotoulas G. (2015). Metagenomics: tools and insights for analyzing next-generation sequencing data derived from biodiversity studies. *Bioinform. Biol. Insights* 9 75–88. 10.4137/BBI.S12462 25983555 PMC4426941

[B112] Pan-Hou H. S. K. Imura N. (1981). Role of hydrogen sulfide in mercury resistance determined by plasmid of *Clostridium* cochlearium T-2. *Arch. Microbiol.* 129 49–52. 10.1007/BF00417179 7224780

[B113] Parks D. H. Chuvochina M. Chaumeil P. A. Rinke C. Mussig A. J. Hugenholtz P. (2020). A complete domain-to-species taxonomy for Bacteria and *Archaea*. *Nat. Biotechnol.* 38 1079–1086. 10.1038/s41587-020-0501-8 32341564

[B114] Parks D. H. Rinke C. Chuvochina M. Chaumeil P.-A. Woodcroft B. J. Evans P. N. (2017). Recovery of nearly 8,000 metagenome-assembled genomes substantially expands the tree of life. *Nat. Microbiol.* 2 1533–1542. 10.1038/s41564-017-0012-7 28894102

[B115] Parks J. M. Johs A. Podar M. Bridou R. Hurt R. A. Smith S. D. (2013). The genetic basis for bacterial mercury methylation. *Science* 339 1332–1335. 10.1126/science.1230667 23393089

[B116] Pei J. Kim B. H. Grishin N. V. (2008). PROMALS3D: a tool for multiple protein sequence and structure alignments. *Nucleic Acids Res.* 36 2295–2300. 10.1093/nar/gkn072 18287115 PMC2367709

[B117] Pickhardt P. C. Fisher N. S. (2007). Accumulation of inorganic and methylmercury by freshwater phytoplankton in two contrasting water bodies. *Environ. Sci. Technol.* 41 125–131. 10.1021/es060966w 17265937

[B118] Pitts K. E. Summers A. O. (2002). The roles of thiols in the bacterial organomercurial lyase (MerB). *Biochemistry* 41 10287–10296. 10.1021/bi0259148 12162744

[B119] Plugge C. M. Zhang W. Scholten J. C. M. Stams A. J. M. (2011). Metabolic flexibility of sulfate-reducing bacteria. *Front. Microbiol.* 2:81. 10.3389/fmicb.2011.00081 21734907 PMC3119409

[B120] Podar M. Gilmour C. C. Brandt C. C. Soren A. Brown S. D. Crable B. R. (2015). Global prevalence and distribution of genes and microorganisms involved in mercury methylation. *Sci. Adv.* 1:e1500675. 10.1126/sciadv.1500675 26601305 PMC4646819

[B121] Poudel S. Colman D. R. Fixen K. R. Ledbetter R. N. Zheng Y. Pence N. (2018). Electron transfer to nitrogenase in different genomic and metabolic backgrounds. *J. Bacteriol.* 200:e757-17. 10.1128/JB.00757-17 29483165 PMC5915786

[B122] Ravichandran M. (2004). Interactions between mercury and dissolved organic matter - a review. *Chemosphere* 55 319–331. 10.1016/j.chemosphere.2003.11.011 14987930

[B123] Rennex D. Cummings R. T. Pickett M. Walsh C. T. Bradley M. (1993). Role of tyrosine residues in mercury(II) detoxification by mercuric reductase from *Bacillus* sp. strain RC607. *Biochemistry* 32 7475–7478. 10.1021/bi00080a019 8338845

[B124] Reysenbach A. (2015). “*Thermoprotei* class. nov,” in *Bergey’s Manual of Systematics of Archaea and Bacteria*, eds Trujillo M. E. Dedysh S. Hedlund P. DeVos B. Kämpfer P. Rainey F. A. (Hoboken, NJ: Wiley), 10.1002/9781118960608.cbm00018

[B125] Rimmer C. C. Miller E. K. McFarland K. P. Taylor R. J. Faccio S. D. (2010). Mercury bioaccumulation and trophic transfer in the terrestrial food web of a montane forest. *Ecotoxicology* 19 697–709. 10.1007/s10646-009-0443-x 19960247

[B126] Rooney J. P. K. (2007). The role of thiols, dithiols, nutritional factors and interacting ligands in the toxicology of mercury. *Toxicology* 234 145–156. 10.1016/j.tox.2007.02.016 17408840

[B127] Roy G. Karri R. Das R. Rai R. K. Gopalakrishnan A. (2020). Hg–C bond protonolysis by a functional model of bacterial enzyme organomercurial lyase MerB. *Chem. Commun.* 56 9280–9283. 10.1039/d0cc02232b 32558833

[B128] Rozov R. Kav A. B. Bogumil D. Shterzer N. Halperin E. Mizrahi I. (2017). Recycler: an algorithm for detecting plasmids from de novo assembly graphs. *Bioinformatics* 33 475–482. 10.1093/bioinformatics/btw651 28003256 PMC5408804

[B129] Sakoula D. Nowka B. Spieck E. Daims H. Lücker S. (2018). The draft genome sequence of “Nitrospira lenta” strain BS10, a nitrite oxidizing bacterium isolated from activated sludge. *Stand. Genomic Sci.* 13:32. 10.1186/s40793-018-0338-7 30498561 PMC6251164

[B130] Sayed A. Ghazy M. A. Ferreira A. J. S. Setubal J. C. Chambergo F. S. Ouf A. (2014). A novel mercuric reductase from the unique deep brine environment of Atlantis II in the Red Sea. *J. Biol. Chem.* 289 1675–1687. 10.1074/jbc.M113.493429 24280218 PMC3894346

[B131] Schartup A. T. Balcom P. H. Soerensen A. L. Gosnell K. J. Calder R. S. D. Mason R. P. (2015). Freshwater discharges drive high levels of methylmercury in Arctic marine biota. *Proc. Natl. Acad. Sci. U. S. A.* 112 11789–11794. 10.1073/pnas.1505541112 26351688 PMC4586882

[B132] Schelert J. Dixit V. Hoang V. Simbahan J. Drozda M. Blum P. (2004). Occurrence and characterization of mercury resistance in the hyperthermophilic archaeon *Sulfolobus solfataricus* by use of gene disruption. *J. Bacteriol.* 186 427–437. 10.1128/JB.186.2.427-437.2004 14702312 PMC305765

[B133] Selin N. E. Javob D. J. Park R. J. Yantosca R. M. Strode S. Jaeglé L. (2007). Chemical cycling and deposition of atmospheric mercury: global constraints from observations. *J. Geophys. Res. Atmos.* 112:14. 10.1029/2006JD007450

[B134] Shelobolina E. S. Nevin K. P. Blakeney-Hayward J. D. Johnsen C. V. Plaia T. W. Krader P. (2007). *Geobacter pickeringii* sp. nov., *Geobacter argillaceus* sp. nov. and *Pelosinus fermentans* gen. nov., sp. nov., isolated from subsurface kaolin lenses. *Int. J. Syst. Evol. Microbiol.* 57 126–135. 10.1099/ijs.0.64221-0 17220454

[B135] Shi L. D. Chen Y. S. Du J. J. Hu Y. Q. Shapleigh J. P. Zhao H. P. (2019). Metagenomic evidence for a Methylocystis species capable of bioremediation of diverse heavy metals. *Front. Microbiol.* 10:3297. 10.3389/fmicb.2018.03297 30687279 PMC6333641

[B136] Sievers F. Wilm A. Dineen D. Gibson T. J. Karplus K. Li W. (2011). Fast, scalable generation of high-quality protein multiple sequence alignments using Clustal Omega. *Mol. Syst. Biol.* 7:539. 10.1038/msb.2011.75 21988835 PMC3261699

[B137] Silver S. Hobman J. L. (2007). “Mercury microbiology: resistance systems, environmental aspects, methylation, and human health,” in *Molecular Microbiology of Heavy Metals*, eds Nies D. H. Silver S. (Berlin: Springer Berlin Heidelberg), 357–370. 10.1007/7171_2006_085

[B138] Simbahan J. Kurth E. Schelert J. Dillman A. Moriyama E. Jovanovich S. (2005). Community analysis of a mercury hot spring supports occurrence of domain-specific forms of mercuric reductase. *Appl. Environ. Microbiol.* 71 8836–8845. 10.1128/AEM.71.12.8836-8845.2005 16332880 PMC1317467

[B139] Slobodkina G. B. Reysenbach A.-L. Panteleeva A. N. Kostrikina N. A. Wagner I. D. Bonch-Osmolovskaya E. A. (2012). *Deferrisoma camini* gen. nov., sp. nov., a moderately thermophilic, dissimilatory iron(III)-reducing bacterium from a deep-sea hydrothermal vent that forms a distinct phylogenetic branch in the *Deltaproteobacteria*. *Int. J. Syst. Evol. Microbiol.* 62 2463–2468. 10.1099/ijs.0.038372-0 22140176

[B140] Song Y. Adediran G. A. Jiang T. Hayama S. Björn E. Skyllberg U. (2020). Toward an internally consistent model for Hg(II) chemical speciation calculations in bacterium-natural organic matter-low molecular mass thiol systems. *Environ. Sci. Technol.* 54 8094–8103. 10.1021/acs.est.0c01751 32491838 PMC7467648

[B141] Stanisich V. A. Bennett P. M. Richmond M. H. (1977). Characterization of a translocation unit encoding resistance to mercuric ions that occurs on a nonconjugative plasmid in *Pseudomonas aeruginosa*. *J. Bacteriol.* 129 1227–1233. 10.1128/JB.129.3.1227-1233.1977 403173 PMC235085

[B142] Stothard P. (2000). The sequence manipulation suite: javascript programs for analyzing and formatting protein and DNA sequences. *Biotechniques* 28 1102–1104. 10.2144/00286ir01 10868275

[B143] Sugio T. Fujii M. Takeuchi F. Negishi A. Maeda T. Kamimura K. (2003). Volatilization of mercury by an iron oxidation enzyme system in a highly mercury-resistant *Acidithiobacillus ferrooxidans* strain mon-1. *Biosci. Biotechnol. Biochem.* 67 1537–1544. 10.1271/bbb.67.1537 12913298

[B144] Sunderland E. M. (2007). Mercury exposure from domestic and imported estuarine and marine fish in the U.S. seafood market. *Environ. Health Perspect.* 115 235–242. 10.1289/ehp.9377 17384771 PMC1817718

[B145] Takaki Y. Shimamura S. Nakagawa S. Fukuhara Y. Horikawa H. Ankai A. (2010). Bacterial lifestyle in a deep-sea hydrothermal vent chimney revealed by the genome sequence of the thermophilic bacterium *Deferribacter desulfuricans* SSM1. *DNA Res.* 17 123–137. 10.1093/dnares/dsq005 20189949 PMC2885270

[B146] Tan S. Liu J. Fang Y. Hedlund B. P. Lian Z. H. Huang L. Y. (2019). Insights into ecological role of a new deltaproteobacterial order Candidatus Acidulodesulfobacterales by metagenomics and metatranscriptomics. *ISME J.* 13 2044–2057. 10.1038/s41396-019-0415-y 30962514 PMC6776010

[B147] Taubner L. M. McGuirl M. A. Dooley D. M. Copié V. (2006). Structural studies of Apo Nosl, an accessory protein of the nitrous oxide reductase system: insights from structural homology with MerB, a mercury resistance protein. *Biochemistry* 45 12240–12252. 10.1021/bi061089 17014077

[B148] Thomas S. A. Catty P. Hazemann J. L. Michaud-Soret I. Gaillard J. F. (2019). The role of cysteine and sulfide in the interplay between microbial Hg(II) uptake and sulfur metabolism. *Metallomics* 11 1219–1229. 10.1039/c9mt00077a 31143907

[B149] Thomas S. A. Rodby K. E. Roth E. W. Wu J. Gaillard J. F. (2018). Spectroscopic and microscopic evidence of biomediated HgS species formation from Hg(II)-cysteine complexes: implications for Hg(II) bioavailability. *Environ. Sci. Technol.* 52 10030–10039. 10.1021/acs.est.8b01305 30078312

[B150] Tomazini A. Higasi P. Manzine L. R. Stott M. Sparling R. Levin D. B. (2019). A novel thermostable GH5 β-xylosidase from Thermogemmatispora sp. T81. *N. Biotechnol.* 53 57–64. 10.1016/j.nbt.2019.07.002 31299302

[B151] Valko M. Morris H. Cronin M. (2005). Metals, toxicity, and oxidative stress. *Curr. Med. Chem.* 12 1161–1208. 10.2174/0929867053764635 15892631

[B152] Vandieken V. Knoblauch C. Jørgensen B. B. (2006). *Desulfovibrio frigidus* sp. nov. and *Desulfovibrio ferrireducens* sp. nov., psychrotolerant bacteria isolated from Arctic fjord sediments (Svalbard) with the ability to reduce Fe(III). *Int. J. Syst. Evol. Microbiol.* 56 681–685. 10.1099/ijs.0.64057-0 16585676

[B153] Varekamp J. C. Buseck P. R. (1986). Global mercury flux from volcanic and geothermal sources. *Appl. Geochemistry* 1 65–73. 10.1016/0883-2927(86)90038-7

[B154] Vetriani C. Chew Y. S. Miller S. M. Yagi J. Coombs J. Lutz R. A. (2005). Mercury adaptation among bacteria from a deep-sea hydrothermal vent. *Appl. Environ. Microbiol.* 71 220–226. 10.1128/AEM.71.1.220-226.2005 15640191 PMC544242

[B155] Vishnivetskaya T. A. Hu H. Van Nostrand J. D. Wymore A. M. Xu X. Qiu G. (2018). Microbial community structure with trends in methylation gene diversity and abundance in mercury-contaminated rice paddy soils in Guizhou, China. *Environ. Sci. Process. Impacts* 20 673–685. 10.1039/c7em00558j 29504614

[B156] Wahba H. M. Lecoq L. Stevenson M. Mansour A. Cappadocia L. Lafrance-Vanasse J. (2016). Structural and biochemical characterization of a copper-binding mutant of the organomercurial lyase MerB: insight into the key role of the active site aspartic acid in Hg-carbon bond cleavage and metal binding specificity. *Biochemistry* 55 1070–1081. 10.1021/acs.biochem.5b01298 26820485

[B157] Wang Y. Moore M. Levinson H. S. Silver S. Walsh C. Mahler I. (1989). Nucleotide sequence of a chromosomal mercury resistance determinant from a *Bacillus* sp. with broad-spectrum mercury resistance. *J. Bacteriol.* 171 83–92. 10.1128/jb.171.1.83-92.1989 2536669 PMC209558

[B158] Watanabe M. Kojima H. Fukui M. (2016). Complete genome sequence and cell structure of *Limnochorda pilosa*, a Gram-negative spore-former within the phylum *Firmicutes*. *Int. J. Syst. Evol. Microbiol.* 66 1330–1339. 10.1099/ijsem.0.000881 26743010

[B159] Watras C. J. Back R. C. Halvorsen S. Hudson R. J. M. Morrison K. A. Wente S. P. (1998). Bioaccumulation of mercury in pelagic freshwater food webs. *Sci. Total Environ.* 219 183–208. 10.1016/S0048-9697(98)00228-9 9802248

[B160] Watras C. J. Morrison K. A. Host J. S. Bloom N. S. (1995). Concentration of mercury species in relationship to other site-specific factors in the surface waters of northern Wisconsin lakes. *Limnol. Oceanogr.* 40 556–565. 10.4319/lo.1995.40.3.0556

[B161] Weber K. A. Achenbach L. A. Coates J. D. (2006). Microorganisms pumping iron: anaerobic microbial iron oxidation and reduction. *Nat. Rev. Microbiol.* 4 752–764. 10.1038/nrmicro1490 16980937

[B162] Wiatrowski H. A. Das S. Kukkadapu R. Ilton E. S. Barkay T. Yee N. (2009). Reduction of Hg(II) to Hg(0) by magnetite. *Environ. Sci. Technol.* 43 5307–5313. 10.1021/es9003608 19708358

[B163] Wiatrowski H. A. Ward P. M. Barkay T. (2006). Novel reduction of mercury(II) by mercury-sensitive dissimilatory metal reducing bacteria. *Environ. Sci. Technol.* 40 6690–6696. 10.1021/es061046g 17144297

[B164] Yu H. Leadbetter J. R. (2020). Bacterial chemolithoautotrophy via manganese oxidation. *Nature* 583 453–458. 10.1038/s41586-020-2468-5 32669693 PMC7802741

[B165] Yu Q. Fein J. B. (2017). Enhanced removal of dissolved Hg(II), Cd(II), and Au(III) from water by Bacillus subtilis bacterial biomass containing an elevated concentration of sulfhydryl sites. *Environ. Sci. Technol.* 51 14360–14367. 10.1021/acs.est.7b04784 29154538

[B166] Zaremba-Niedzwiedzka K. Caceres E. F. Saw J. H. Bäckström D. Juzokaite L. Vancaester E. (2017). Asgard *Archaea* illuminate the origin of eukaryotic cellular complexity. *Nature* 541 353–358. 10.1038/nature21031 28077874

[B167] Zhalnina K. V. Dias R. Leonard M. T. Dorr de Quadros P. Camargo F. A. O. Drew J. C. (2014). Genome sequence of *Candidatus Nitrososphaera* evergladensis from group I.1b enriched from Everglades soil reveals novel genomic features of the ammonia-oxidizing *Archaea*. *PLoS One* 9:e101648. 10.1371/journal.pone.0101648 24999826 PMC4084955

[B168] Zhang H. Feng X. Larssen T. Qiu G. Vogt R. D. (2010). In Inland China, rice, rather than fish, is the major pathway for methylmercury exposure. *Environ. Health Perspect.* 118 1183–1188. 10.1289/ehp.1001915 20378486 PMC2944075

[B169] Zheng Y. Saitou A. Wang C. M. Toyoda A. Minakuchi Y. Sekiguchi Y. (2019). Genome features and secondary metabolites biosynthetic potential of the class Ktedonobacteria. *Front. Microbiol.* 10:893. 10.3389/fmicb.2019.00893 31080444 PMC6497799

[B170] Zillig W. Reysenbach A. (2015). “*Thermococci* class. nov.,” in *Bergey’s Manual of Systematics of Archaea and Bacteria*, eds M. E. Trujillo, S. Dedysh, P. DeVos, B. Hedlund, P. Kämpfer, F. A. Rainey, et al. (Hoboken, NJ: Wiley), 10.1002/9781118960608.cbm00030

[B171] Zolnikov T. R. Ramirez Ortiz D. (2018). A systematic review on the management and treatment of mercury in artisanal gold mining. *Sci. Total Environ.* 633 816–824. 10.1016/j.scitotenv.2018.03.241 29602119

