# Supplementary material for: Expanded Diversity and Phylogeny of mer Genes Broadens Mercury Resistance Paradigms and Reveals an Origin for MerA Among Thermophilic Archaea
Source: Front Microbiol. 2021 Jun 23;12:682605. doi: 10.3389/fmicb.2021.682605 (PMC8261052; doi:10.3389/fmicb.2021.682605)
Supplement: Supplementary file 1 [file Data_Sheet_1.ZIP › Supplemental Figures.pdf]

Supplemental Figures.

A

B

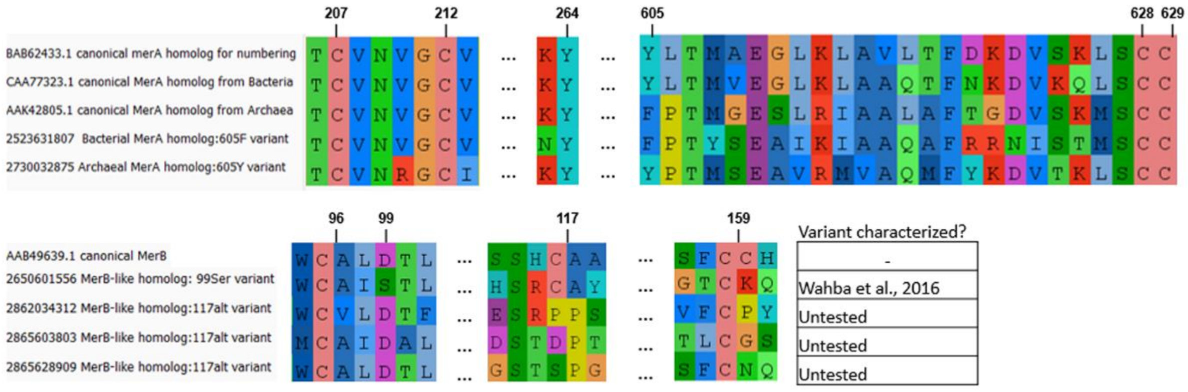

Supplementary Figure 1. Canonical MerA and MerB homologs and their variants. A) MerA homolog (top) and select MerA homolog variants (bottom four sequences). B) MerB homolog (top) and select MerB homolog variants (bottom four sequences). Amino acid positions depicted are based on MerA from *Bacillus cereus* (BAB62433; panel A) and MerB from Escherichia coli (AAB49639; panel B).

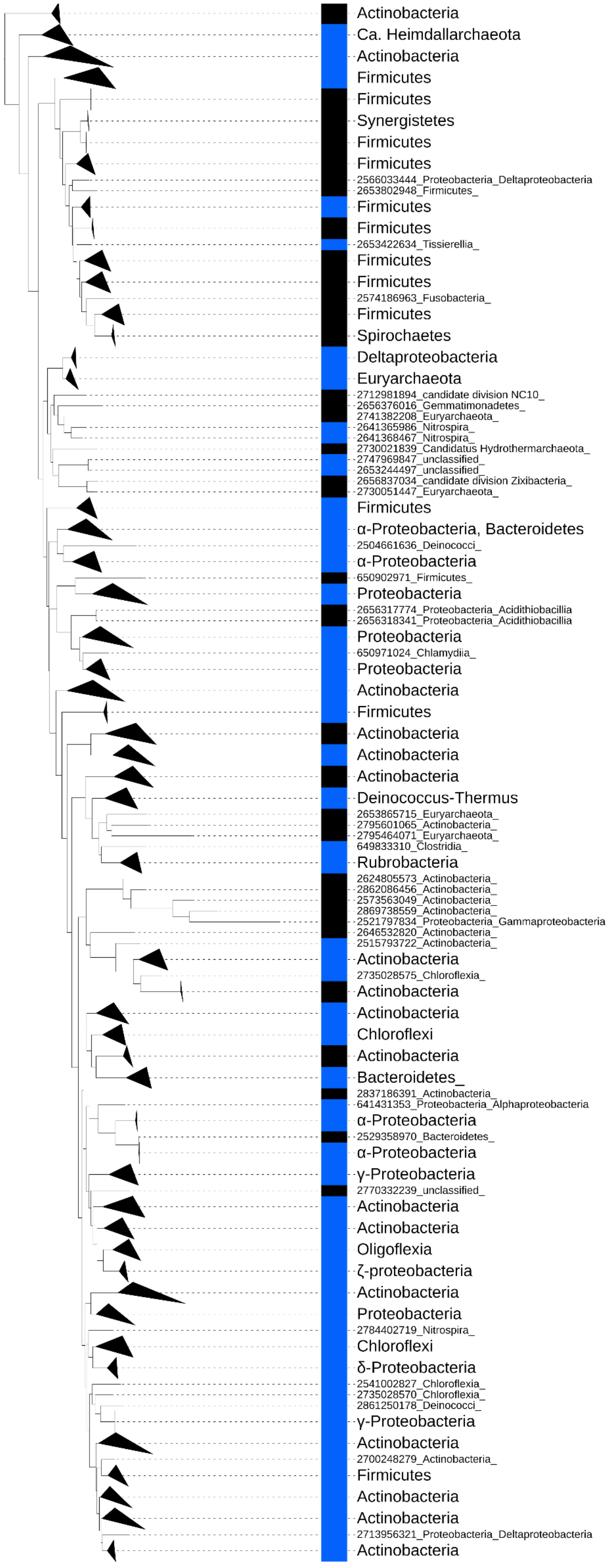

Supplementary Figure 2. Phylogenetic of MerB protein homologs (plasmid MerB not included). The outer boxes to the right of labels show the presence (blue boxes) and absence of MerA (black boxes) homologs in the genomes from which the MerB homologs were derived. Branch length is in amino acid substitutions per site.
